# TreeSummarizedExperiment: a S4 class for data with hierarchical structure

**DOI:** 10.12688/f1000research.26669.2

**Published:** 2021-03-02

**Authors:** Ruizhu Huang, Charlotte Soneson, Felix G.M. Ernst, Kevin C. Rue-Albrecht, Guangchuang Yu, Stephanie C. Hicks, Mark D. Robinson

**Affiliations:** 1 Department of Molecular Life Sciences, University of Zurich, Zurich, Switzerland; 2 SIB Swiss Institute of Bioinformatics, Zurich, Switzerland; 3 Friedrich Miescher Institute for Biomedical Research, Basel, Switzerland; 4 Population Health Sciences, German Center for Neurodegenerative Diseases (DZNE), Bonn, Germany; 5 MRC WIMM Centre for Computational Biology, University of Oxford, Oxford, OX3 9DS, UK; 6 Department of Bioinformatics, School of Basic Medical University, Guangzhou, Guangdong, China; 7 Microbiome Medicine Center, Division of Laboratory Medicine, Zhujiang Hospital, Southern Medical University, Guangzhou, Guangdong, China; 8 Department of Biostatistics, Johns Hopkins Bloomberg School of Public Health, Baltimore, USA

**Keywords:** SummarizedExperiment, tree, microbiome, hierarchical structure

## Abstract

Data organized into hierarchical structures (e.g., phylogenies or cell types) arises in several biological fields. It is therefore of interest to have data containers that store the hierarchical structure together with the biological profile data, and provide functions to easily access or manipulate data at different resolutions. Here, we present TreeSummarizedExperiment, a R/S4 class that extends the commonly used SingleCellExperiment class by incorporating tree representations of rows and/or columns (represented by objects of the phylo class). It follows the convention of the SummarizedExperiment class, while providing links between the assays and the nodes of a tree to allow data manipulation at arbitrary levels of the tree. The package is designed to be extensible, allowing new functions on the tree (phylo) to be contributed. As the work is based on the SingleCellExperiment class and the phylo class, both of which are popular classes used in many R packages, it is expected to be able to interact seamlessly with many other tools.

## Introduction

Biological data arranged into a hierarchy occurs in several fields. A notable example is in microbial survey studies, where the microbiome is profiled with amplicon sequencing or whole genome shotgun sequencing, and microbial taxa are organized as a tree according to their similarities in the genomic sequence or the evolutionary history. Also, a tree might be used in single cell cytometry or RNA-seq data, with nodes representing cell subpopulations at different granularities ^1^. Currently, phyloseq ^2^ and ***SingleCellExperiment*** ^3^ are popular classes used in the analysis of microbial data and single cell data, respectively. The former supports the information pertaining to the hierarchical structure that is available as the phylo class (e.g., phylogenetic tree), and the latter is derived from the ***SummarizedExperiment*** class (defined in the *SummarizedExperiment* package ^4^), which is widely used as a standardized container across many Bioconductor packages. Since the data structures in these fields share similarities, we were motivated to develop an S4 class ^5^, ***TreeSummarizedExperiment***, that not only leverages the facilities from the ***SummarizedExperiment*** class, but also bridges the functionality from the phylo class, which is available from the *ape* ^6^ package and has been imported in more than 200 R packages.

We define ***TreeSummarizedExperiment*** by extending the ***SingleCellExperiment*** class, so that it is a member of the ***SummarizedExperiment*** family, and thus benefits from the comprehensive Bioconductor ecosystem (e.g., *iSEE* ^7^, *SEtools* ^8^, and *ggbio* ^9^). At the same time, all slots of the phyloseq class have their corresponding slots in the ***TreeSummarizedExperiment*** class, which enables convenient conversion between these classes. Furthermore, we allow the link between profile data and nodes of the tree, including leaves and internal nodes, which is useful for algorithms in the downstream analysis that need to access internal nodes of the tree (e.g., treeclimbR ^1^).

Overall, the class ***TreeSummarizedExperiment*** is provided as a standalone R package, analogous to ***SummarizedExperiment*** and ***SingleCellExperiment***. Thus, it is convenient for R package developers to import it and build downstream data analyses or visualizations on it. Also, it is flexible to combine with R packages that are linked to the ***SummarizedExperiment*** family or the phylo tree class, which enables R package users to explore data with the support of other tools.

## Methods

### Implementation

**Figure 1.**
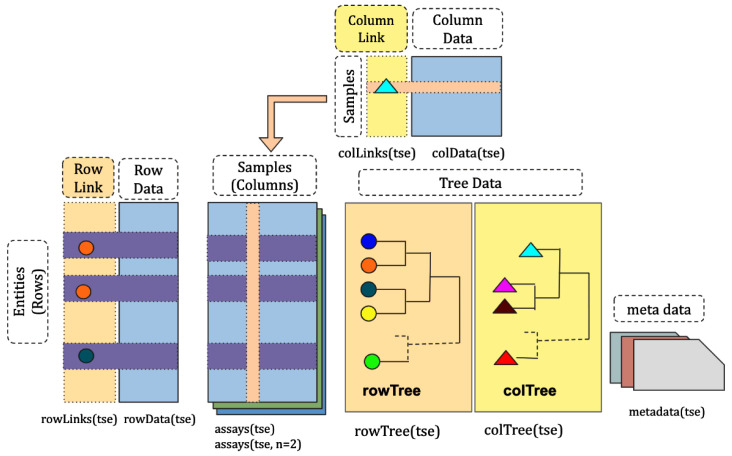
The structure of the *TreeSummarizedExperiment* class. The rectangular data matrices are stored in assays. Each matrix usually has rows representing entities (e.g., genes or microbial taxa) and columns representing cells or samples. Information about rows and columns is stored in rowData and colData, respectively. The hierarchy structure on rows or columns is stored in rowTree or colTree respectively, and the link information between rows/columns and nodes of the row/column tree is in rowLinks/colLinks. referenceSeq is an optional slot to store the sequence information for rows.

***The structure of TreeSummarizedExperiment***. The structure of the ***TreeSummarizedExperiment*** class is shown in Figure 1.

Compared to the ***SingleCellExperiment*** objects, ***TreeSummarizedExperiment*** has five additional slots:

- rowTree: the hierarchical structure on the rows of the assays.
- rowLinks: the link information between rows of the assays and the rowTree.
- colTree: the hierarchical structure on the columns of the assays.
- colLinks: the link information between columns of the assays and the colTree.
- referenceSeq (optional): the sequence information for rows of the assays.

The rowTree and */*or colTree can be left empty ( NULL) if no trees are available; in this case, the rowLinks and */*or colLinks are also set to NULL. The referenceSeq is an optional slot to store the sequence data of features either as DNAStringSet or DNAStringSetList. All other ***TreeSummarizedExperiment*** slots are inherited from ***SingleCellExperiment***.

The rowTree and colTree slots require the tree to be an object of the phylo class. If a tree is available in an alternative format, it can often be converted to a phylo object using dedicated R packages (e.g., *treeio* ^10^).

Functions in the *TreeSummarizedExperiment* package fall in two main categories: operations on the ***TreeSummarizedExperiment*** object or operations on the tree ( phylo) objects. The former includes constructors and accessors, and the latter serves as “components” to be assembled as accessors or functions that manipulate the ***TreeSummarizedExperiment*** object. Given that more than 200 R packages make use of the phylo class, there are many resources (e.g., *ape*) for users to manipulate the small “pieces” in addition to those provided in *TreeSummarizedExperiment*. 

### The toy datasets as the example data

We generate a toy dataset that has observations of 6 entities collected from 4 samples as an example to show how to construct a ***TreeSummarizedExperiment*** object.


library(TreeSummarizedExperiment)

# assays data (typically, representing observed data from an experiment)
assay_data <- rbind(rep(0, 4), matrix(1:20, nrow = 5))
colnames(assay_data) <- paste0("sample", 1:4)
rownames(assay_data) <- paste0("entity", seq_len(6))
assay_data



##         sample1 sample2 sample3 sample4
## entity1       0       0       0       0
## entity2       1       6      11      16
## entity3       2       7      12      17
## entity4       3       8      13      18
## entity5       4       9      14      19
## entity6       5      10      15      20


The information of entities and samples are given in the **row_data** and **col_data**, respectively.


# row data (feature annotations)
row_data <- data.frame(Kingdom = "A",
                          Phylum = rep(c("B1", "B2"), c(2, 4)),
                          Class = rep(c("C1", "C2", "C3"), each = 2),
                          OTU = paste0("D", 1:6),
                          row.names = rownames(assay_data),
                          stringsAsFactors = FALSE)
row_data



##         Kingdom Phylum Class OTU
## entity1       A     B1    C1  D1
## entity2       A     B1    C1  D2
## entity3       A     B2    C2  D3
## entity4       A     B2    C2  D4
## entity5       A     B2    C3  D5
## entity6       A     B2    C3  D6



# column data (sample annotations)
col_data <- data.frame(gg = c(1, 2, 3, 3),
                          group = rep(LETTERS[1:2], each = 2),
                          row.names = colnames(assay_data),
                          stringsAsFactors = FALSE)
col_data

##         gg group
## sample1  1     A
## sample2  2     A
## sample3  3     B
## sample4  3     B


The hierarchical structure of the 6 entities and 4 samples are denoted as **row_tree** and **col_tree**, respectively. The two trees are phylo objects randomly created with rtree from the package *ape*. Note that the row tree has 5 rather than 6 leaves; this is used later to show that multiple rows in the assays are allowed to map to a single node in the tree.


library(ape)

# The first toy tree
set.seed(12)
row_tree <- rtree(5)

# The second toy tree
set.seed(12)
col_tree <- rtree(4)

# change node labels
col_tree$tip.label <- colnames(assay_data)
col_tree$node.label <- c("All", "GroupA", "GroupB")


**Figure 2.**
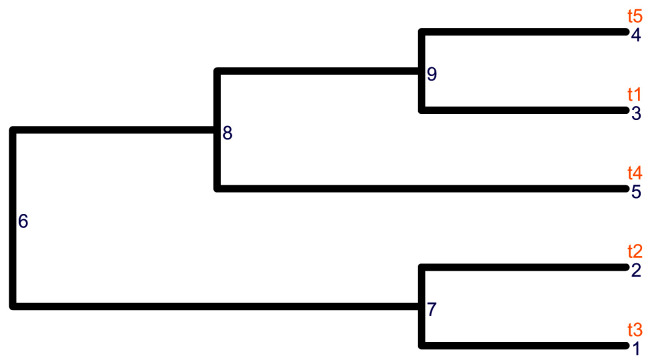
The structure of the row tree. The node labels and the node numbers are in orange and blue text, respectively.

We visualize the tree using the package *ggtree* (v. 2.2.4) ^11^. Here, the internal nodes of the **row_tree** have no labels as shown in Figure 2.


library(ggtree)
library(ggplot2)

# Visualize the row tree
ggtree(row_tree, size = 2, branch.length = "none") +
     geom_text2(aes(label = node), color = "darkblue",
                 hjust = -0.5, vjust = 0.7, size = 4) +
     geom_text2(aes(label = label), color = "darkorange",
                 hjust = -0.1, vjust = -0.7, size = 4)


The **col_tree** has labels for internal nodes as shown in Figure 3.


# Visualize the column tree
ggtree(col_tree, size = 2, branch.length = "none") +
     geom_text2(aes(label = node), color = "darkblue",
                 hjust = -0.5, vjust = 0.7, size = 4) +
     geom_text2(aes(label = label), color = "darkorange",
                 hjust = -0.1, vjust = -0.7, size = 4)+
     ylim(c(0.8, 4.5)) +
     xlim(c(0, 2.2))


**Figure 3.**
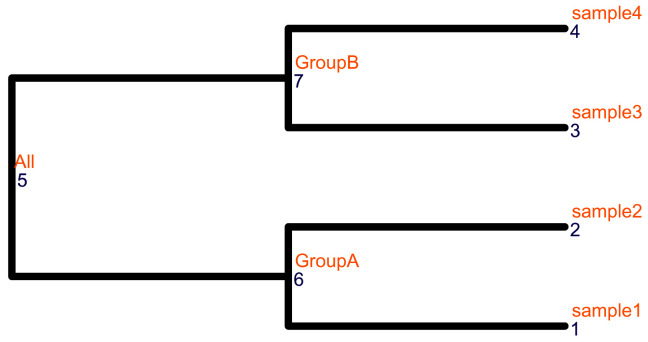
The structure of the column tree. The node labels and the node numbers are in orange and blue text, respectively.

### The construction of *TreeSummarizedExperiment*

The ***TreeSummarizedExperiment*** class is used to store the toy data generated in the previous section: **assay_data**, **row_data**, **col_data**, **col_tree** and **row_tree**. To correctly store data, the link information between the rows (or columns) of **assay_data** and the nodes of the **row_tree** (or **col_tree**) can be provided via a character vector rowNodeLab (or colNodeLab), with length equal to the number of rows (or columns) of the assays; otherwise the row (or column) names are used. Tree data takes precedence to determine entities included during the creation of the ***TreeSummarizedExperiment*** object; columns and rows with labels that are not present among the node labels of the tree are removed with warnings. The link data between the assays tables and the tree data is automatically generated during the construction.

The row and column trees can be included simultaneously during the construction of a ***TreeSummarized-Experiment*** object. Here, the column names of **assay_data** can be found in the node labels of the column tree, which enables the link to be created between the column dimension of **assay_data** and the column tree **col_tree**. If the row names of **assay_data** are not in the node labels of **row_tree**, we would need to provide their corresponding node labels ( **row_lab**) to rowNodeLab in the construction of the object. It is possible to map multiple rows or columns to a node, for example, the same leaf label is used for the first two rows in **row_lab**.


# all column names could be found in the node labels of the column tree
all(colnames(assay_data) %in% c(col_tree$tip.label, col_tree$node.label))



## [1] TRUE



# provide the node labels in rowNodeLab
tip_lab <- row_tree$tip.label
row_lab <- tip_lab[c(1, 1:5)]
row_lab



## [1] "t3" "t3" "t2" "t1" "t5" "t4"



both_tse <- TreeSummarizedExperiment(assays = list(Count = assay_data),
                                         rowData = row_data,
                                         colData = col_data,
                                         rowTree = row_tree,
                                         rowNodeLab = row_lab,
                                         colTree = col_tree)



both_tse

## class: TreeSummarizedExperiment
## dim: 6 4
## metadata(0):
## assays(1): Count
## rownames(6): entity1 entity2 ... entity5 entity6
## rowData names(4): Kingdom Phylum Class OTU
## colnames(4): sample1 sample2 sample3 sample4
## colData names(2): gg group
## reducedDimNames(0):
## altExpNames(0):
## rowLinks: a LinkDataFrame (6 rows)
## rowTree: 1 phylo tree(s) (5 leaves)
## colLinks: a LinkDataFrame (4 rows)
## colTree: 1 phylo tree(s) (4 leaves)


When printed on screen, ***TreeSummarizedExperiment*** objects display information as the parent ***SingleCell-Experiment*** class followed by four additional lines for rowLinks, rowTree, colLinks and colTree.

### The accessor functions

Slots inherited from the ***SummarizedExperiment*** class can be accessed in the standard way (e.g., via accessors assays(), rowData(), colData() and metadata()). These functions are both getters and setters. To clarify, getters and setters are functions for users to retrieve and to overwrite data from the corresponding slots, respectively. Here, accessors for ***TreeSummarizedExperiment*** are both getters and setters unless specifically mentioned.

For new slots, we provide rowTree (and colTree) to access the row (column) trees, and rowLinks (and colLinks) as getters to retrieve the link information between assays and the row (column) tree. If the tree is not available, the corresponding link data is NULL.


# access trees
rowTree(both_tse)
##
## Phylogenetic tree with 5 tips and 4 internal nodes.
##
## Tip labels:
##   t3, t2, t1, t5, t4
##
## Rooted; includes branch lengths.



colTree(both_tse)



##
## Phylogenetic tree with 4 tips and 3 internal nodes.
##
## Tip labels:
##   sample1, sample2, sample3, sample4
## Node labels:
##   All, GroupA, GroupB
##
## Rooted; includes branch lengths.



# access the link data
(r_link <- rowLinks(both_tse))



## LinkDataFrame with 6 rows and 5 columns
##             nodeLab nodeLab_alias   nodeNum    isLeaf   whichTree
##         <character>   <character> <integer> <logical> <character>
## entity1          t3       alias_1         1      TRUE       phylo
## entity2          t3       alias_1         1      TRUE       phylo
## entity3          t2       alias_2         2      TRUE       phylo
## entity4          t1       alias_3         3      TRUE       phylo
## entity5          t5       alias_4         4      TRUE       phylo
## entity6          t4       alias_5         5      TRUE       phylo



(c_link <- colLinks(both_tse))



## LinkDataFrame with 4 rows and 5 columns
##             nodeLab nodeLab_alias   nodeNum    isLeaf   whichTree
##         <character>   <character> <integer> <logical> <character>
## sample1     sample1       alias_1         1      TRUE       phylo
## sample2     sample2       alias_2         2      TRUE       phylo
## sample3     sample3       alias_3         3      TRUE       phylo
## sample4     sample4       alias_4         4      TRUE       phylo


The link data objects are of the LinkDataFrame class, which extends the DataFrame class from *S4Vectors* with the restriction that it has five columns:

- nodeLab: the labels of nodes on the tree
- nodeLab_alias: the alias labels of nodes on the tree
- nodeNum: the numbers of nodes on the tree
- isLeaf: whether the node is a leaf node
- whichTree: which tree the row/col is linked to

The data in colLinks() is updated automatically with the change of colTree().


# remove the column tree
colTree(both_tse) <- NULL

# the colLinks() is updated accordingly
colLinks(both_tse)

## NULL

# colTree works as a setter
colTree(both_tse) <- col_tree
colLinks(both_tse)

## LinkDataFrame with 4 rows and 5 columns
##       nodeLab   nodeNum nodeLab_alias    isLeaf   whichTree
##   <character> <integer>   <character> <logical> <character>
## 1     sample1         1       alias_1      TRUE       phylo
## 2     sample2         2       alias_2      TRUE       phylo
## 3     sample3         3       alias_3      TRUE       phylo
## 4     sample4         4       alias_4      TRUE       phylo


### The subsetting function

A ***TreeSummarizedExperiment*** object can be subset in two different ways: [ to subset by rows or columns, and subsetByNode to retrieve row and/or columns that correspond to nodes of a tree. As the numeric ID of a node changes with the cut of a phylo tree, to keep track of the original data, we do not prune the tree structure in the subsetting. Below, we can see that rowLinks and rowData are updated to have the same number of rows as assays.


sub_tse <- both_tse[1:2, 1]
sub_tse

## class: TreeSummarizedExperiment
## dim: 2 1
## metadata(0):
## assays(1): Count
## rownames(2): entity1 entity2
## rowData names(4): Kingdom Phylum Class OTU
## colnames(1): sample1
## colData names(2): gg group
## reducedDimNames(0):
## altExpNames(0):
## rowLinks: a LinkDataFrame (2 rows)
## rowTree: 1 phylo tree(s) (5 leaves)
## colLinks: a LinkDataFrame (1 rows)
## colTree: 1 phylo tree(s) (4 leaves)



# the row data
rowData(sub_tse)

## DataFrame with 2 rows and 4 columns
##             Kingdom      Phylum       Class         OTU
##         <character> <character> <character> <character>
## entity1           A          B1          C1          D1
## entity2           A          B1          C1          D2



# the row link data
rowLinks(sub_tse)

## LinkDataFrame with 2 rows and 5 columns
##             nodeLab nodeLab_alias   nodeNum    isLeaf   whichTree
##         <character>   <character> <integer> <logical> <character>
## entity1          t3       alias_1         1      TRUE       phylo
## entity2          t3       alias_1         1      TRUE       phylo



# The first four columns are from colLinks data and the others from colData
cbind(colLinks(sub_tse), colData(sub_tse))

## DataFrame with 1 row and 7 columns
##       nodeLab   nodeNum nodeLab_alias    isLeaf   whichTree        gg
##   <character> <integer>   <character> <logical> <character> <numeric>
## 1     sample1         1       alias_1      TRUE       phylo         1
##         group
##   <character>
## 1           A


To subset by nodes, we specify the node by its node label or node number. Here, *entity1* and *entity2* are both mapped to the same node t3, so both of them are retained.


node_tse <- subsetByNode(x = both_tse, rowNode = "t3")

rowLinks(node_tse)

## LinkDataFrame with 2 rows and 5 columns
##             nodeLab nodeLab_alias   nodeNum    isLeaf   whichTree
##         <character>   <character> <integer> <logical> <character>
## entity1          t3       alias_1         1      TRUE       phylo
## entity2          t3       alias_1         1      TRUE       phylo


Subsetting simultaneously in both dimensions is also allowed.


node_tse <- subsetByNode(x = both_tse, rowNode = "t3",
                            colNode = c("sample1", "sample2"))
assays(node_tse)[[1]]

##         sample1 sample2
## entity1       0       0
## entity2       1       6


### Changing the tree

The current tree can be replaced by a new one using changeTree. Here, we don’t use rowTree() to do the replacement because the new tree has node labels that cannot match with row names of the ***TreeSummarizedExperiment*** object. If the hierarchical information is available as a data.frame with each column representing a taxonomic level (e.g., *row_data*), we provide toTree to convert it into a phylo object that is further visualized in Figure 4.


# The toy taxonomic table
(taxa <- rowData(both_tse))

## DataFrame with 6 rows and 4 columns
##             Kingdom      Phylum       Class         OTU
##         <character> <character> <character> <character>
## entity1           A          B1          C1          D1
## entity2           A          B1          C1          D2
## entity3           A          B2          C2          D3
## entity4           A          B2          C2          D4
## entity5           A          B2          C3          D5
## entity6           A          B2          C3          D6


**Figure 4.**
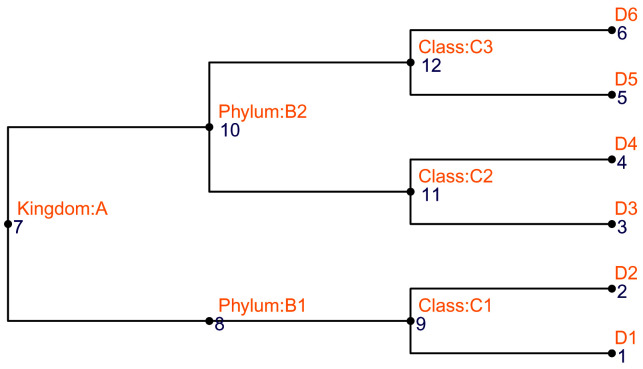
The structure of the taxonomic tree that is generated from the taxonomic table.


# convert it to a phylo tree
taxa_tree <- toTree(data = taxa)

# Viz the new tree
ggtree(taxa_tree)+
     geom_text2(aes(label = node), color = "darkblue",
                 hjust = -0.5, vjust = 0.7, size = 4) +
     geom_text2(aes(label = label), color = "darkorange",
                 hjust = -0.1, vjust = -0.7, size = 4) +
     geom_point2()


If the nodes of the two trees have a different set of labels, a vector mapping the nodes of the new tree must be provided in rowNodeLab.


taxa_tse <- changeTree(x = both_tse, rowTree = taxa_tree,
                          rowNodeLab = taxa[["OTU"]])

taxa_tse



## class: TreeSummarizedExperiment
## dim: 6 4
## metadata(0):
## assays(1): Count
## rownames(6): entity1 entity2 ... entity5 entity6
## rowData names(4): Kingdom Phylum Class OTU
## colnames(4): sample1 sample2 sample3 sample4
## colData names(2): gg group
## reducedDimNames(0):
## altExpNames(0):
## rowLinks: a LinkDataFrame (6 rows)
## rowTree: 1 phylo tree(s) (6 leaves)
## colLinks: a LinkDataFrame (4 rows)
## colTree: 1 phylo tree(s) (4 leaves)



rowLinks(taxa_tse)

## LinkDataFrame with 6 rows and 4 columns
##             nodeLab nodeLab_alias   nodeNum    isLeaf   whichTree
##         <character>   <character> <integer> <logical> <character>
## entity1          D1       alias_1         1      TRUE       phylo
## entity2          D2       alias_2         2      TRUE       phylo
## entity3          D3       alias_3         3      TRUE       phylo
## entity4          D4       alias_4         4      TRUE       phylo
## entity5          D5       alias_5         5      TRUE       phylo
## entity6          D6       alias_6         6      TRUE       phylo


### Aggregation

Since it may be of interest to report or analyze observed data at multiple resolutions based on the provided tree(s), the ***TreeSummarizedExperiment*** package offers functionality to flexibly aggregate data to arbitrary levels of a tree.

**The column dimension.** Here, we demonstrate the aggregation functionality along the column dimension. The desired aggregation level is given in the colLevel argument, which can be specified using node labels (orange text in Figure 3) or node numbers (blue text in Figure 3). Furthermore, the summarization method used to aggregate multiple values can be specified via the argument colFun.


# use node labels to specify colLevel
agg_col <- aggTSE(x = taxa_tse,
                    colLevel = c("GroupA", "GroupB"),
                    colFun = sum)
# or use node numbers to specify colLevel
agg_col <- aggTSE(x = taxa_tse, colLevel = c(6, 7), colFun = sum)


assays(agg_col)[[1]]

##         alias_6 alias_7
## entity1       0       0
## entity2       7      27
## entity3       9      29
## entity4      11      31
## entity5      13      33
## entity6      15      35


The rowData does not change, but the colData is updated to reflect the metadata information that remains valid for the individual nodes after aggregation. For example, the column **group** has the A value for GroupA because the descendant nodes of GroupA all have the value A; whereas the column **gg** has the NA value for GroupA because the descendant nodes of GroupA have different values, (1 and 2).


# before aggregation
colData(taxa_tse)



## DataFrame with 4 rows and 2 columns
##                gg       group
##         <numeric> <character>
## sample1         1           A
## sample2         2           A
## sample3         3           B
## sample4         3           B



# after aggregation
colData(agg_col)



## DataFrame with 2 rows and 2 columns
##                gg       group
##         <numeric> <character>
## alias_6        NA           A
## alias_7         3           B


The colLinks is also updated to link the new rows of assays tables to the corresponding nodes of the column tree ( Figure 3).


# the link data is updated
colLinks(agg_col)

## LinkDataFrame with 2 rows and 5 columns
##             nodeLab nodeLab_alias   nodeNum    isLeaf   whichTree
##         <character>   <character> <integer> <logical> <character>
## alias_6      GroupA       alias_6         6     FALSE       phylo
## alias_7      GroupB       alias_7         7     FALSE       phylo


**The row dimension.** Similarly, we can aggregate rows to phyla by providing the names of the internal nodes that represent the phylum level (see taxa_one below).


# the phylum level
taxa <- c(taxa_tree$tip.label, taxa_tree$node.label)
(taxa_one <- taxa[startsWith(taxa, "Phylum:")])



## [1] "Phylum:B1" "Phylum:B2"



# aggregation
agg_taxa <- aggTSE(x = taxa_tse, rowLevel = taxa_one, rowFun = sum)
assays(agg_taxa)[[1]] 

##          sample1 sample2 sample3 sample4
## alias_8        1       6      11      16
## alias_10      14      34      54      74


Users are nonetheless free to choose nodes from different taxonomic ranks for each final aggregated row. Note that it is not necessary to use all original rows during the aggregation process. Similarly, it is entirely possible for a row to contribute to multiple aggregated rows.


# A mixed level
taxa_mix <- c("Class:C3", "Phylum:B1")
agg_any <- aggTSE(x = taxa_tse, rowLevel = taxa_mix, rowFun = sum)
rowData(agg_any)



## DataFrame with 2 rows and 4 columns
##              Kingdom      Phylum       Class       OTU
##          <character> <character> <character> <logical>
## alias_12           A          B2          C3        NA
## alias_8            A          B1          C1        NA


**Both dimensions.** The aggregation on both dimensions could be performed in one step, in which case users can specify the order of aggregation; either rows first (rowFirst = TRUE) or columns first (rowFirst = FALSE). The aggregate functions for the row and the column dimension can be provided via rowFun and colFun, respectively. Additionally, parallel computation is enable by providing BPPARAM with a BiocParallelParam object.


agg_both <- aggTSE(x = both_tse,
                     rowLevel = 7:9, rowFun = sum,
                     colLevel = 6:7, colFun = mean,
                     rowFirst = FALSE)


As expected, we obtain a table with 3 rows representing the aggregated row nodes 7, 8 and 9 ( rowLevel = 7:9) and 2 columns representing the aggregated column nodes 6 and 7 ( colLevel = 6:7).


assays(agg_both)[[1]]

##         alias_6 alias_7
## alias_7     8.0    28.0
## alias_8    19.5    49.5
## alias_9    12.0    32.0


### Functions operating on the phylo object

Next, we highlight some functions to manipulate and/or to extract information from the phylo object. Further operations can be found in other packages, such as *ape* ^6^, *tidytree* ^12^. These functions are useful for users who wish to develop more functions for the ***TreeSummarizedExperiment*** class.

**Figure 5.**
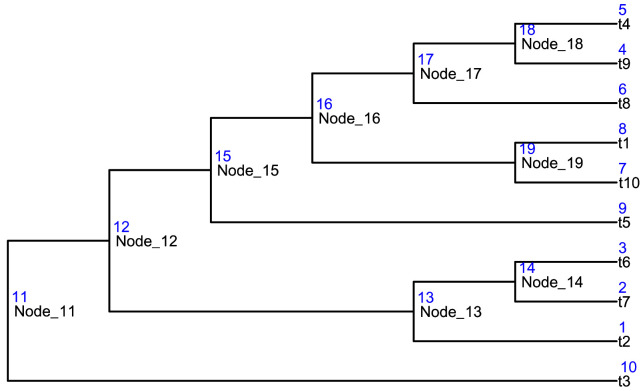
An example tree with node labels and numbers in black and blue texts, respectively.

To show these functions, we use the tree shown in Figure 5.


data("tinyTree")
ggtree(tinyTree, branch.length = "none") +
     geom_text2(aes(label = label), hjust = -0.1, size = 3) +
     geom_text2(aes(label = node), vjust = -0.8,
                 hjust = -0.2, color = "blue", size = 3)


**Conversion of the node label and the node number** The translation between the node labels and node numbers can be achieved by the function convertNode.


convertNode(tree = tinyTree, node = c(12, 1, 4))



## [1] "Node_12" "t2"      "t9"



convertNode(tree = tinyTree, node = c("t4", "Node_18"))



##      t4 Node_18
##       5      18


**Find the descendants** To get descendants that are at the leaf level, we could set the argument only.leaf = TRUE for the function findDescendant.


# only the leaf nodes
findDescendant(tree = tinyTree, node = 17, only.leaf = TRUE)



## $Node_17
## [1] 4 5 6


When only.leaf = FALSE, all descendants are returned.


# all descendant nodes
findDescendant(tree = tinyTree, node = 17, only.leaf = FALSE)

## $Node_17
## [1]  4  5  6 18


**More functions.** We list some functions that might also be useful in Table 1. More functions are available in the package, and we encourage users to develop and contribute their own functions to the package.

**Table 1.** A table lists some functions operating on the phylo object that are available in the *TreeSummarizedExperiment*.

Functions	Goal
printNode	print out the information of nodes
countNode	count the number of nodes
distNode	give the distance between a pair of nodes
matTree	list paths of a tree
findAncestor	find ancestor nodes
findChild	find child nodes
findSibling	find sibling nodes
shareNode	find the first node shared in the paths of nodes to the root
unionLeaf	find the union of descendant leaves
trackNode	track nodes by adding alias labels to a phylo object
isLeaf	test whether a node is a leaf node
showNode	print out nodes of a tree
addLabel	label nodes of a tree
joinNode	represent descendant nodes with their ancestor nodes

### Custom functions for the *TreeSummarizedExperiment* class

Here, we show examples of how to write custom functions for ***TreeSummarizedExperiment*** objects. To extract data corresponding to specific leaves, we created a function subsetByLeaf by combining functions working on the phylo class (e.g., convertNode, keep.tip, trackNode) with the accessor function subsetByNode. Here, convertNode and trackNode are available in ***TreeSummarizedExperiment***, and keep.tip is from the *ape* package. Since the numeric identifier of a node is changed after pruning a tree with keep.tip, trackNode is provided to track the node and further update links between the rectangular assay matrices and the new tree.


# tse: a TreeSummarizedExperiment object
# rowLeaf: specific leaves
subsetByLeaf <- function(tse, rowLeaf) {
  # if rowLeaf is provided as node labels, convert them to node numbers
  if (is.character(rowLeaf)) {
    rowLeaf <- convertNode(tree = rowTree(tse), node = rowLeaf)
  }

  # subset data by leaves
  sse <- subsetByNode(tse, rowNode = rowLeaf)

  # update the row tree
    ## -------------- new tree: drop leaves ----------
    oldTree <- rowTree(sse)
    newTree <- ape::keep.tip(phy = oldTree, tip = rowLeaf)

    ## -------------- update the row tree ----------
    # track the tree
    track <- trackNode(oldTree)
    track <- ape::keep.tip(phy = track, tip = rowLeaf)

    # update the row tree:
    #   1. get the old alias label and update it to the new node label
    #   2. provide the new node label as rowNodeLab to update the row tree
    oldAlias <- rowLinks(sse)$nodeLab_alias
    newNode <- convertNode(tree = track, node = oldAlias)
    newLab <- convertNode(tree = newTree, node = newNode)

    changeTree(x = sse, rowTree = newTree, rowNodeLab = newLab)
}


The row tree is updated; after subsetting, it has only two leaves, t2 and t3.


(both_sse <- subsetByLeaf(tse = both_tse, rowLeaf = c("t2", "t3")))



## class: TreeSummarizedExperiment
## dim: 3 4
## metadata(0):
## assays(1): Count
## rownames(3): entity1 entity2 entity3
## rowData names(4): Kingdom Phylum Class OTU
## colnames(4): sample1 sample2 sample3 sample4
## colData names(2): gg group
## reducedDimNames(0):
## altExpNames(0):
## rowLinks: a LinkDataFrame (3 rows)
## rowTree: 1 phylo tree(s) (2 leaves)
## colLinks: a LinkDataFrame (4 rows)
## colTree: 1 phylo tree(s) (4 leaves)



rowLinks(both_sse)

## LinkDataFrame with 3 rows and 5 columns
##             nodeLab nodeLab_alias   nodeNum    isLeaf   whichTree
##         <character>   <character> <integer> <logical> <character>
## entity1          t3       alias_1         1      TRUE       phylo
## entity2          t3       alias_1         1      TRUE       phylo
## entity3          t2       alias_2         2      TRUE       phylo


### Operation

The ***TreeSummarizedExperiment*** package can be installed by following the standard installation procedures of Bioconductor packages.


# install BiocManager
if (!requireNamespace("BiocManager", quietly = TRUE))
     install.packages("BiocManager")

# install TreeSummarizedExperiment package
BiocManager::install("TreeSummarizedExperiment")


Minimum system requirements is R version 3.6 (or later) on a Mac, Windows or Linux system. The version of ***TreeSummarizedExperiment*** should be later than 1.99.10, which is available in Bioconductor 3.13.

## Use cases

### HMP 16S rRNA-seq data

To demonstrate the functionality of ***TreeSummarizedExperiment***, we use it to store and manipulate a microbial dataset. We further show exploratory graphics using the available functions designed for ***SummarizedExperiment*** objects in other packages (e.g., *scater*), or customized functions from popular visualization packages (e.g., *ggplot2* ^13^).


# Packages providing dataset
library(HMP16SData)

# Package to do parallel computation
library(BiocParallel)

# Packages to manipulate data extracted from TreeSummarizedExperiment
library(tidyr)
library(dplyr)



# Packages providing visualization
library(ggplot2)
library(scales)
library(ggtree)
library(scater)
library(cowplot)


The Human Microbiome Project (HMP) 16S rRNA sequencing data, v35, is downloaded using the R package *HMP16SData* ^14^, which contains survey data of samples collected at five major body sites in the variable regions 3–5. v35 is available as a SummarizedExperiment object via the ExperimentHub.


(v35 <- V35())

## class: SummarizedExperiment
## dim: 45383 4743
## metadata(2): experimentData phylogeneticTree
## assays(1): 16SrRNA
## rownames(45383): OTU_97.1 OTU_97.10 ... OTU_97.9998 OTU_97.9999
## rowData names(7): CONSENSUS_LINEAGE SUPERKINGDOM ... FAMILY GENUS
## colnames(4743): 700013549 700014386 ... 700114717 700114750
## colData names(7): RSID VISITNO ... HMP_BODY_SUBSITE SRS_SAMPLE_ID

# name the assay
names(assays(v35)) <- "Count"


### The storage of HMP 16S rRNA-seq data

We store the phylogenetic tree as the rowTree. Links between nodes of the tree and rows of assays are automatically generated in the construction of the ***TreeSummarizedExperiment*** object, and are stored as rowLinks. Rows of the assays matrices that do not have a match to nodes of the tree are removed with warnings.


(tse_phy <- TreeSummarizedExperiment(assays = assays(v35),
                                         rowData = rowData(v35),
                                         colData = colData(v35),
                                         rowTree = metadata(v35)$phylogeneticTree,
                                         metadata = metadata(v35)["experimentData"]))


## Warning: 47 row(s) couldn’t be matched to the tree and are/is removed.

## class: TreeSummarizedExperiment
## dim: 45336 4743
## metadata(1): experimentData
## assays(1): Count
## rownames(45336): OTU_97.1 OTU_97.10 ... OTU_97.9998 OTU_97.9999
## rowData names(7): CONSENSUS_LINEAGE SUPERKINGDOM ... FAMILY GENUS
## colnames(4743): 700013549 700014386 ... 700114717 700114750
## colData names(7): RSID VISITNO ... HMP_BODY_SUBSITE SRS_SAMPLE_ID
## reducedDimNames(0):
## altExpNames(0):
## rowLinks: a LinkDataFrame (45336 rows)
## rowTree: 1 phylo tree(s) (45364 leaves)
## colLinks: NULL
## colTree: NULL

cD <- colData(tse_phy)
dim(table(cD$HMP_BODY_SITE, cD$RUN_CENTER))

## [1] 5 12


### Exploratory graphics

Here, we show ***TreeSummarizedExperiment*** working seamlessly with *SEtools* (v.1.2.0) to prepare data for the exploratory graphics. Since all operational taxonomic units (OTUs) in the sample belong to Bacteria in the SUPERKINGDOM level, we can calculate the sequencing depths by aggregating counts to the SUPERKINGDOM level. The resultant ***TreeSummarizedExperiment*** object **agg_total** is further converted into a data frame **df_total** with selected columns ( HMP_BODY_SITE and RUN_CENTER) from the column data.


library(SEtools)
agg_total <- aggSE(x = tse_phy, by = "SUPERKINGDOM",
                     assayFun = sum)

# The assays data and selected columns of the row/col data are merged into a
# data frame
df_total <- meltSE(agg_total, genes = rownames(agg_total),
                     colDat.columns = c("HMP_BODY_SITE", "RUN_CENTER"))

head(df_total)

##    feature    sample          HMP_BODY_SITE RUN_CENTER Count
## 1 Bacteria 700013549 Gastrointestinal Tract        BCM  5295
## 2 Bacteria 700014386 Gastrointestinal Tract     BCM,BI 10811
## 3 Bacteria 700014403                   Oral     BCM,BI 12312
## 4 Bacteria 700014409                   Oral     BCM,BI 20355
## 5 Bacteria 700014412                   Oral     BCM,BI 14021
## 6 Bacteria 700014415                   Oral     BCM,BI 17157


To make harmonized figures with ggplot2 (v.3.3.2) ^13^, we customized a theme to be applied to several plots in this section.


# Customized the plot theme
prettify <- theme_bw(base_size = 10) + theme(
     panel.spacing = unit(0, "lines"),
     axis.text = element_text(color = "black"),
     axis.text.x = element_text(angle = 45, hjust = 1),
     axis.title = element_text(size = 8),
     legend.key.size= unit(3, "mm"),
     legend.spacing.x = unit(1, "mm"),
     plot.title = element_text(hjust = 0.5),
     legend.text = element_text(size = 8),
     legend.position="bottom",
     strip.background = element_rect(colour = "black", fill = "gray90"),
     strip.text.x = element_text(color = "black", size = 10),
     strip.text.y = element_text(color = "black", size = 10))


**Figure 6.**
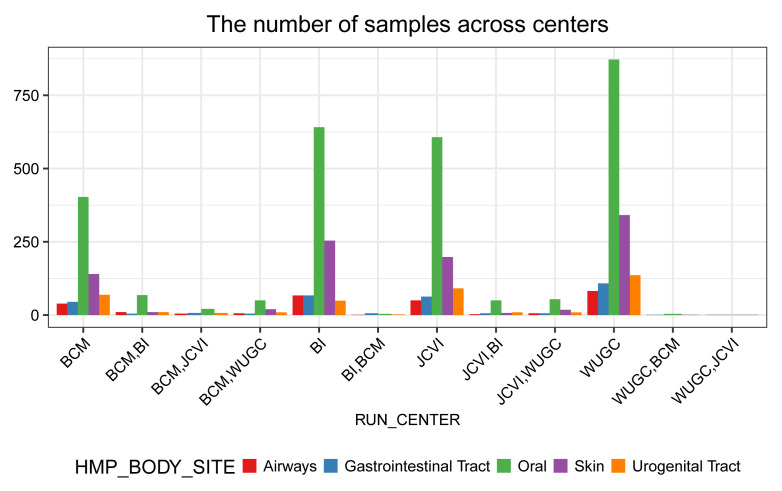
The number of samples from different research centers. Samples collected at different body sites are in different colors.

From Figure 6, we note that more samples were collected from the oral site than other body sites.


# Figure: (the number of samples) VS (centers)
ggplot(df_total) +
     geom_bar(aes(RUN_CENTER, fill = HMP_BODY_SITE),
                position = position_dodge()) +
     labs(title = "The number of samples across centers", y = "") +
     scale_fill_brewer(palette = "Set1") +
     prettify


Figure 7 shows that the sequencing depth of each sample across different coordination centers are quite similar. Within the coordination center, samples collected from Skin are more variable in the sequencing depth than those from other body sites.


# Figure: (the sequencing depths) VS (centers)
ggplot(df_total) +
     geom_boxplot(aes(x = RUN_CENTER, y = Count, fill = HMP_BODY_SITE),
                   position = position_dodge()) +
     labs(title = "The sequencing depths of samples") +
     scale_y_continuous(trans = log10_trans()) +
     scale_fill_brewer(palette = "Set1") +
     labs(y = "Total counts") +
     prettify


**Figure 7.**
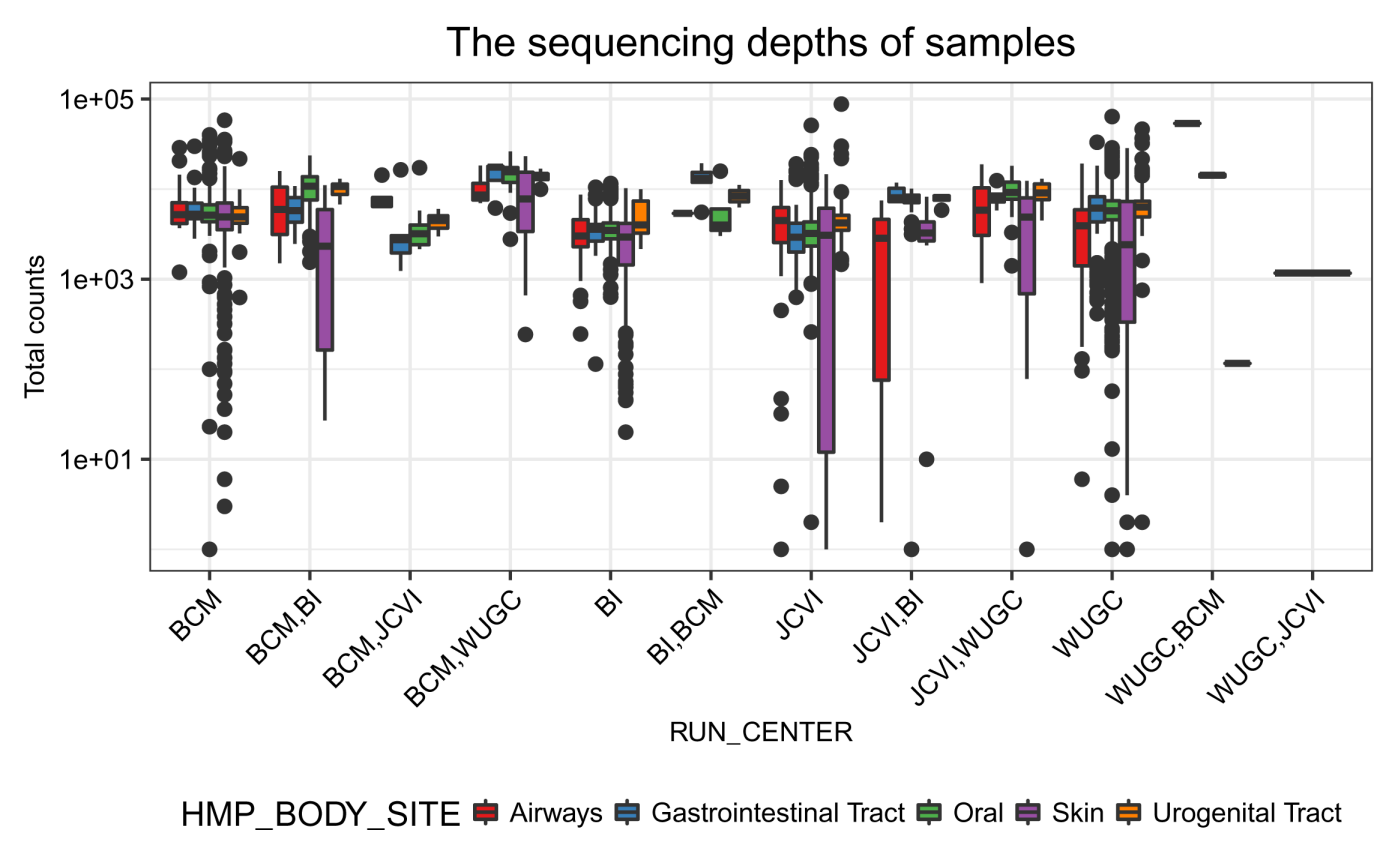
The sequencing depth of samples from different research centers. Samples collected at different body sites are in different colors.

### Dimensionality reduction

We visualize samples in reduced dimensions to see whether those from the same body site are similar to each other. Three dimensionality reduction techniques are available in the package *scater* (v. 1.16.2), including principal component analysis (PCA) ^15^, t-distributed Stochastic Neighbor Embedding (t-SNE) ^16^, and uniform manifold approximation and projection (UMAP) ^17^. Since ***TreeSummarizedExperiment*** extends the ***SingleCellExperiment*** class, functions from *scater* ^18^ can be used directly. Here, we first apply PCA and t-SNE on data at the OTU level, and select the one better clustering the samples to apply on data aggregated at coarser taxonomic levels to see whether the resolution affects the separation of samples.

**PCA and t-SNE at the OTU level** The PCA is performed on the log-transformed counts that are stored in the assays matrix with the name logcounts. In practice, data normalization is usually applied prior to the downstream analysis, to address bias or noise introduced during the sampling or sequencing process (e.g., uneven sampling depth). Here, the library size is highly variable ( Figure 7) and non-zero OTUs vary across body sites. It is difficult to say what is the optimal normalization strategy, and the use of an inappropriate normalization method might introduce new biases. The discussion of normalization is outside the scope of this work. To keep it simple, we will visualize data without further normalization.

**Figure 8.**
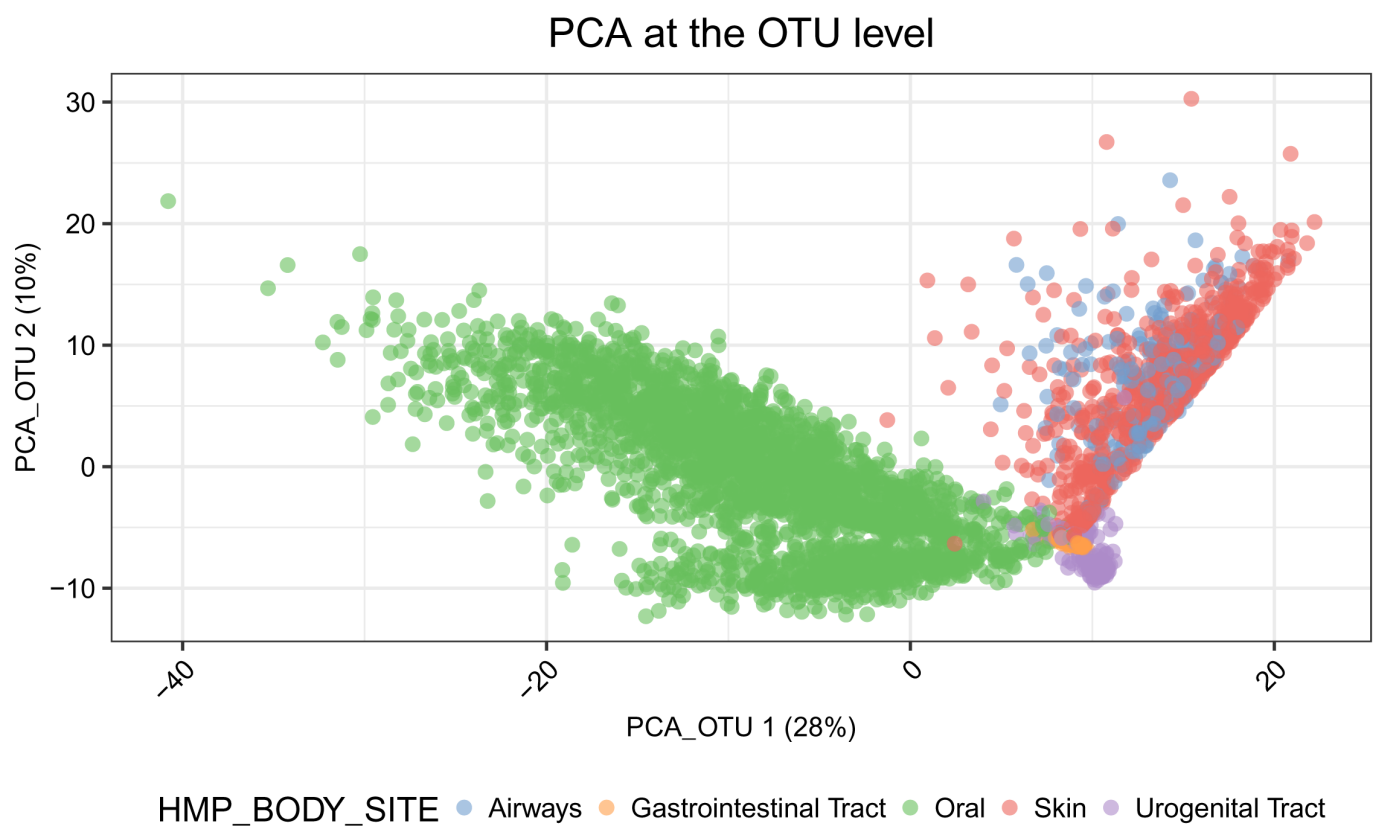
Principal component analysis (PCA) plot of samples using data at the OTU level. The first two principal components (PCs) are plotted. Each point represents a sample. Samples are coloured according to the body sites.

In Figure 8, we see that the Oral samples are distinct from those of other body sites. Samples from Skin, Urogenital Tract, Airways and Gastrointestinal Tract are not separated very well in the first two principal components.


# log-transformed data
assays(tse_phy)$logcounts <- log(assays(tse_phy)$Count + 1)

# run PCA at the OTU level
tse_phy <- runPCA(tse_phy, name="PCA_OTU", exprs_values = "logcounts")

# plot samples in the reduced dimensions
plotReducedDim(tse_phy, dimred = "PCA_OTU",
                 colour_by = "HMP_BODY_SITE")+
     labs(title = "PCA at the OTU level") +
     guides(fill = guide_legend(override.aes = list(size=2.5, alpha = 1))) +
     prettify


**Figure 9.**
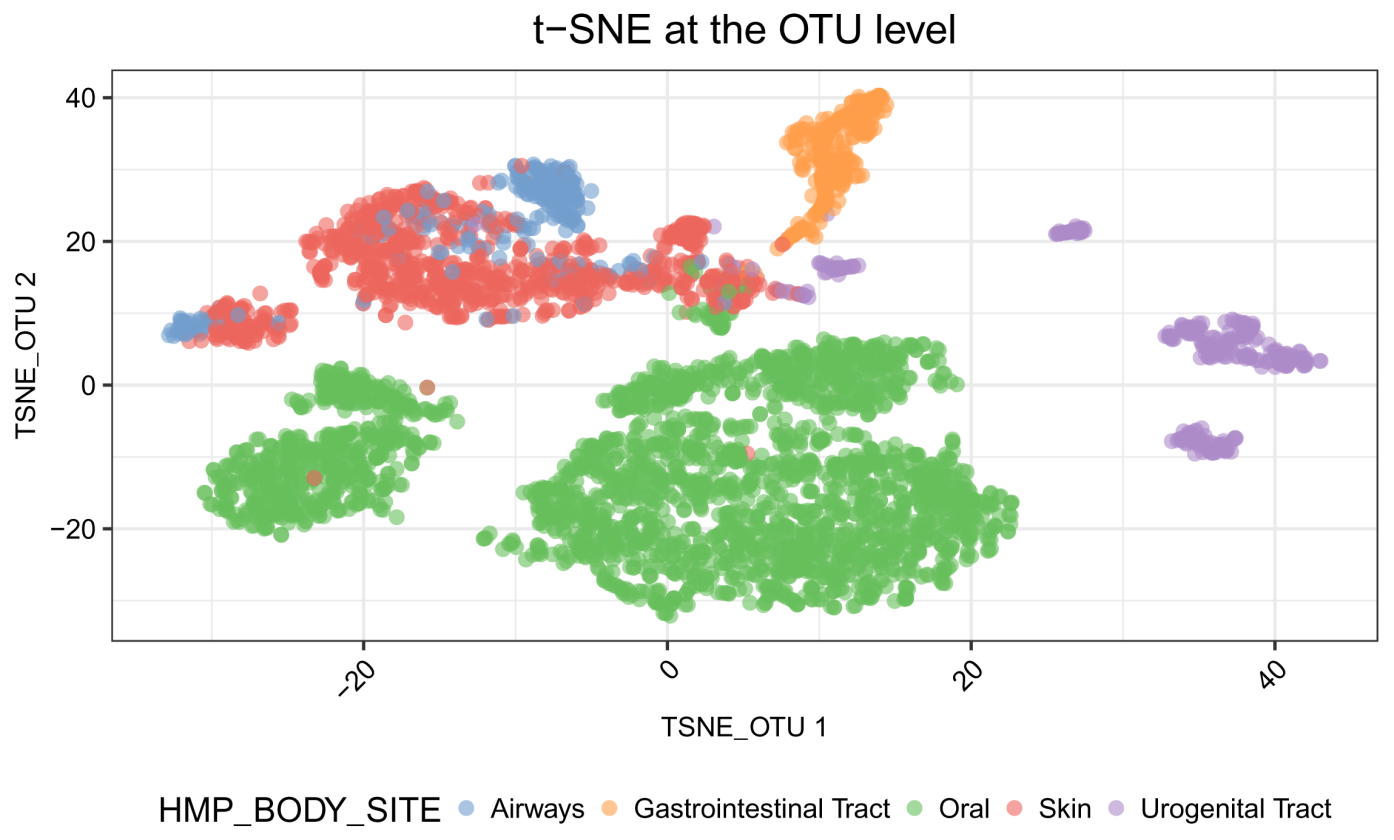
t-SNE plot of samples using data at the OTU level. The first two t-SNE components are plotted. Each point represents a sample. Samples are coloured according to the body site.

The separation is well improved with the use of t-SNE in Figure 9. Samples from Oral, Gastrointestinal Tract, and Urogenital Tract form distinct clusters. Skin samples and airways samples still overlap.


# run t-SNE at the OTU level
tse_phy <- runTSNE(tse_phy, name="TSNE_OTU", exprs_values = "logcounts")

# plot samples in the reduced dimensions
tsne_otu <- plotReducedDim(tse_phy, dimred = "TSNE_OTU",
                               colour_by = "HMP_BODY_SITE") +
     labs(title = "t-SNE at the OTU level") +
     theme(plot.title = element_text(hjust = 0.5)) +     
     scale_fill_brewer(palette = "Set1") +
     labs(fill = "Body sites") +
     guides(fill = guide_legend(override.aes = list(size=2.5, alpha = 1))) +
     prettify
tsne_otu


**Figure 10.**
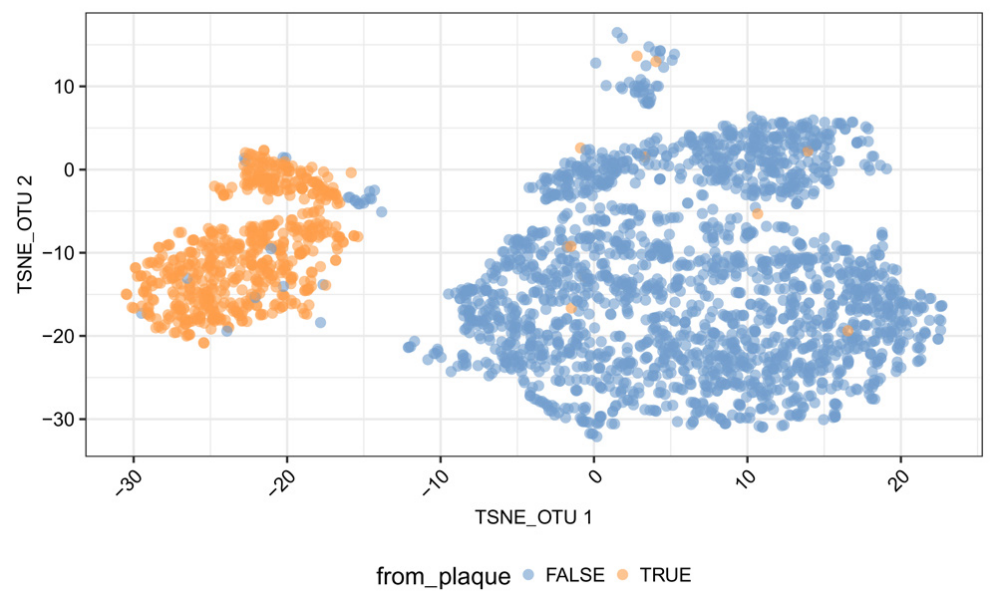
t-SNE plot of samples from the oral site using data at the OTU level. The two t-SNE components computed are plotted. Each point is a sample. Samples from the ‘supragingival or subgingival Plaque‘ are in orange, and those from other oral sub-sites are in blue.

Notably, there are two well-separated clusters labelled as oral samples. The smaller cluster includes samples from the Supragingival Plaque and Subgingival Plaque sites, while the larger cluster includes samples from other oral sub-sites ( Figure 10).


is_oral <- colData(tse_phy)$HMP_BODY_SITE %in% "Oral"
colData(tse_phy)$from_plaque <- grepl(pattern = "Plaque",
                                         colData(tse_phy)$HMP_BODY_SUBSITE)
# Oral samples
plotReducedDim(tse_phy[, is_oral], dimred = "TSNE_OTU",
                 colour_by = "from_plaque") +
  guides(fill = guide_legend(override.aes = list(size=2.5, alpha = 1))) +
  prettify


**t-SNE on broader taxonomic levels** To organize data at different taxonomic levels, we first replace the phylogenetic tree with the taxonomic tree that is generated from the taxonomic table. Due to the existence of polyphyletic groups, a tree structure cannot be generated. For example, the Alteromonadaceae family is from different orders: Alteromonadales and Oceanospirillales.


# taxonomic tree
tax_order <- c("SUPERKINGDOM", "PHYLUM", "CLASS",
                 "ORDER", "FAMILY", "GENUS", "CONSENSUS_LINEAGE")
tax_0 <- data.frame(rowData(tse_phy)[, tax_order])
tax_loop <- detectLoop(tax_tab = tax_0)

# show loops that are not caused by NA
head(tax_loop[!is.na(tax_loop$child), ])



##               parent            child parent_column child_column
## 35   Alteromonadales Alteromonadaceae         ORDER       FAMILY
## 36 Oceanospirillales Alteromonadaceae         ORDER       FAMILY
## 37       Rhizobiales Rhodobacteraceae         ORDER       FAMILY
## 38   Rhodobacterales Rhodobacteraceae         ORDER       FAMILY
## 39      Chromatiales  Sinobacteraceae         ORDER       FAMILY
## 40   Xanthomonadales  Sinobacteraceae         ORDER       FAMILY


To resolve the loops, we add a suffix to the polyphyletic genus with resolveLoop. For example, Ruminococcus belonging to the Lachnospiraceae and the Ruminococcaceae families become Ruminococcus_1 and Ruminococcus_2, respectively. A phylo tree is created afterwards using toTree.


tax_1 <- resolveLoop(tax_tab = tax_0)
tax_tree <- toTree(data = tax_1)

# change the tree
tse_tax <- changeTree(x = tse_phy, rowTree = tax_tree,
                         rowNodeLab = rowData(tse_phy)$CONSENSUS_LINEAGE)


**Figure 11.**
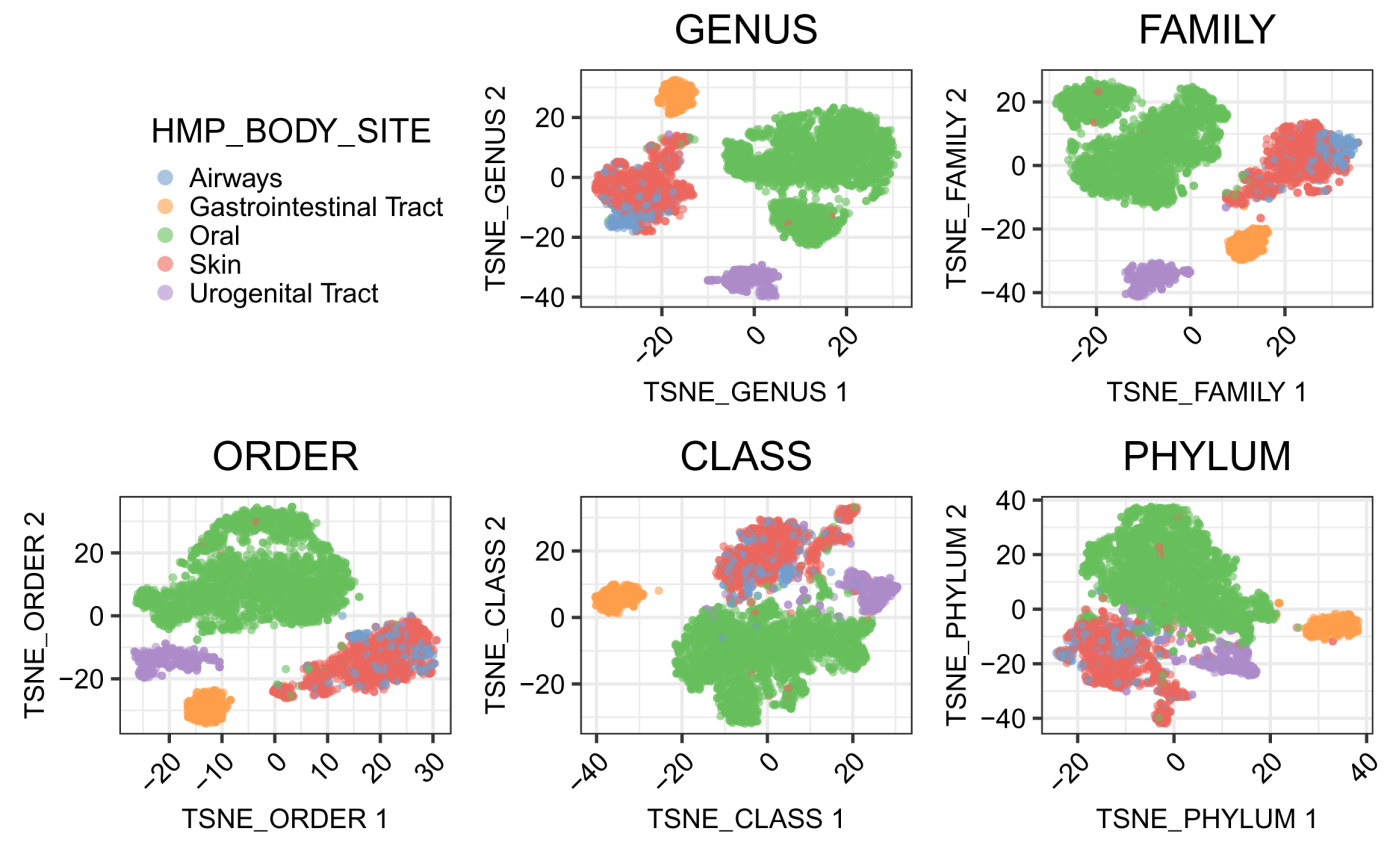
t-SNE plot of samples using data at different taxonomic levels. The two t-SNE components computed are plotted. Each point is a sample. Samples are colored according to the body sites.

The separation of samples from different body sites appears to be worse when the data on broader resolution is used ( Figure 11).


# aggregation data to all internal nodes
# Parallel computation is performed with BPPARAM
tse_agg <- aggTSE(x = tse_tax,
                    rowLevel = tax_tree$node.label,
                    whichAssay = "Count",
                    BPPARAM = MulticoreParam(),
                    message = FALSE)
                       
# log-transform count
assays(tse_agg)$logcounts <- log(assays(tse_agg)[[1]] + 1)


Specifically, we loop over each taxonomic rank and generate a t-SNE representation using data aggregated at that taxonomic rank level.


tax_rank <- c("GENUS", "FAMILY", "ORDER", "CLASS", "PHYLUM")
names(tax_rank) <- tax_rank
fig_list <- lapply(tax_rank, FUN = function(x) {
  # nodes represent the specific taxonomic level
  xx <- startsWith(rowLinks(tse_agg)$nodeLab, x)

  # run t-SNE on the specific level
  xx_tse <- runTSNE(tse_agg, name = paste0("TSNE_", x),
                      exprs_values = "logcounts",
                      subset_row = rownames(tse_agg)[xx])
                       
  # plot samples in the reduced dimensions
  plotReducedDim(xx_tse, dimred = paste0("TSNE_", x),
                   colour_by = "HMP_BODY_SITE",
                   point_size = 0.5) +
    labs(title = x) +
    prettify +
    theme(legend.position = "none")+    
    scale_fill_brewer(palette = "Set1") +
  guides(fill = guide_legend(override.aes = list(size=2.5)))
})



legend <- get_legend(
  # create some space to the left of the legend
  tsne_otu +
    theme(legend.box.margin = margin(0, 0, 0, 35),
           legend.position = "right")
  )
plot_grid(plotlist = fig_list,
           legend, nrow = 2)


### CyTOF data

Here, a mass cytometry (CyTOF) dataset ^19^ is used to show the application of ***TreeSummarizedExperiment*** on single cell data. The data was available initially as a ***SummarizedExperiment*** object, and became a ***TreeSummarizedExperient*** object after the incorporation of a tree on cells. Data was then aggregated along nodes of the tree to provide data at different resolutions. The data visualization was finally performed as heatmaps along with the tree using the R package *ggtree*. 



# packages for visualization
library(ggplot2)
library(ggtree)
library(ggnewscale)
library(RColorBrewer)

# packages for data download and preprocess
library(HDCytoData)
library(diffcyt)
library(ape)

# packages for data manipulation
library(dplyr)
library(tidyr)
library(tibble)


The mass cytometry (CyTOF) dataset ^19^ is downloaded from the R package *HDCytoData* ^20^. The data has 16 samples (eight pairs) of peripheral blood cell collected from eight healthy individuals. Each pair consists of one unstimulated sample, and one sample stimulated with B cell receptor/Fc receptor cross-linker (BCR-XL). The data contains expressions of 24 protein markers: 10 surface lineage markers (type) and 14 intracellular signaling functional markers (state), from 172791 cells.



# download data
d_se <- Bodenmiller_BCR_XL_SE()

# Extract data of protein markers
# surface lineage markers: type
# intracellular signaling functional markers: state
is_ab <- colData(d_se)$marker_class %in% c("type", "state")
d_se <- d_se[, is_ab]
d_se

## class: SummarizedExperiment
## dim: 172791 24
## metadata(2): experiment_info n_cells
## assays(1): exprs
## rownames: NULL
## rowData names(4): group_id patient_id sample_id population_id
## colnames(24): CD3 CD45 ... HLA-DR CD7
## colData names(3): channel_name marker_name marker_class


We preprocess the data and cluster cells using the workflow from the *diffcyt* package ^21, 22^. According to the median expressions of lineage markers per cluster, a tree cytof_hclust is then constructed by applying the hierarchical clustering on the cell cluster level, using only the “type” gene.



# Transform data
d_se <- transformData(d_se)

# Include a random seed to generate a reproducible clustering
d_se <- generateClusters(d_se, xdim = 7, ydim = 7, seed_clustering = 12)
rowData(d_se)$cluster_id <- paste0("cluster_", rowData(d_se)$cluster_id)

# Use cluster IDs as row names
rownames(d_se) <- rowData(d_se)$cluster_id

# Generate a tree with cell clusters as leaves
d_medians <- calcMediansByClusterMarker(d_se)
md <- assay(d_medians)[, metadata(d_medians)$id_type_markers]
cytof_hclust <- hclust(dist(md, method = "manhattan"), method = "mcquitty")


The data d_se is converted from ***SummarizedExperiment*** to ***TreeSummarizedExperiment*** to provide a rowTree slot for the storage the tree information.



# The tree format: convert from hclust to phylo; label internal nodes
cytof_tree <- as.phylo(cytof_hclust)
cytof_tree <- addLabel(tree = cytof_tree, on = "internal")

# Construct a TreeSummarizedExperiment object
lse <- as(d_se, "TreeSummarizedExperiment")
rowTree(lse) <- cytof_tree


In lse, multiple rows (cells) are mapped to a leaf (a cell cluster) of the tree.



# Data corresponding to the cluster_1
subsetByNode(lse, rowNode = "cluster_1")

## class: TreeSummarizedExperiment
## dim: 2461 24
## metadata(3): experiment_info n_cells MST
## assays(1): exprs
## rownames(2461): cluster_1 cluster_1 ... cluster_1 cluster_1
## rowData names(5): group_id patient_id sample_id population_id
##   cluster_id
## colnames(24): CD3 CD45 ... HLA-DR CD7
## colData names(3): channel_name marker_name marker_class
## reducedDimNames(0):
## altExpNames(0):
## rowLinks: a LinkDataFrame (2461 rows)
## rowTree: 1 phylo tree(s) (49 leaves)
## colLinks: NULL
## colTree: NULL


### Data aggregation

We split the data into two ***TreeSummarizedExperiment*** objects: lse_type for lineage markers and lse_state for functional markers, to perform aggregation in different ways. For lse_type, the marker median expression is calculated over all samples to compare expression patterns of lineage markers across all cell clusters. For lse_state, the marker median expression is computed on individual samples, to enable comparison between stimulated and unstimulated samples across clusters.



# Split TSE: lineage markers and functional markers
lse_type <- lse[, colData(lse)$marker_class == "type"]
lse_state <- lse[, colData(lse)$marker_class == "state"]

# All nodes of the tree
nodes <- showNode(tree = cytof_tree, only.leaf = FALSE)
length(nodes)

## [1] 97

# Calculate marker median expressions for clusters
tse_type <- aggTSE(x = lse_type, rowLevel = nodes, rowFun = median)
# use node labels instead of node alias as row names
rownames(tse_type) <- rowLinks(tse_type)$nodeLab
# row --> node; column --> marker
dim(tse_type)

## [1] 97 10

# Calculate marker median expressions for clusters separately on each sample
tse_state <- aggTSE(x = lse_state, rowLevel = nodes, rowFun = median,
                      rowBlock = "sample_id")
# use node labels instead of node alias as row names
rownames(tse_state) <- rowLinks(tse_state)$nodeLab
# row --> node per sample; column --> marker
dim(tse_state)

## [1] 1552 14


After aggregation, tse_type and tse_state have 97 and 1552 rows, respectively. The former has each row representing a cell cluster that is mapped to a node of the tree; the latter has each row representing a cell cluster in a sample.

In the downloaded data, cells are annotated with cell types ( population_id in rowData()). As clustering is not perfect, cells within a cluster are not expected to have exactly the same cell type. Therefore, we would like to annotate a cell cluster with the cell type that the majority of cells (> 60%) belong to, or mixed if none of cell types has more than > 60% cells. Note, internal nodes of the tree cytof_tree are considered as cell clusters at broader resolution than leaf nodes. To annotate an internal node, we need to first find all cells that are mapped to its descendant leaves, and then take the cell type shared by its majority of cells.



# Find descendant leaves of all nodes; Leaves return themselves
desd_leaf <- findDescendant(tree = cytof_tree, node = nodes,
                               only.leaf = TRUE, self.include = TRUE)
# For example, Node_90 has two descendant leaves: 32 & 33 (node number)
desd_leaf[[90]]

## Node_90 Node_90
##      32      33

# Decide cell type for each node according to majorities of cells belong to it
threshold <- 0.6
ct <- sapply(desd_leaf, FUN = function(x) {
  # Data of cells belong to the descendant leaves (x) of a node
  xse <- lse[rowLinks(lse)$nodeNum %in% x, ]

  # Percentages of cell types
  tx <- table(rowData(xse)$population_id)
  pr <- tx/sum(tx)

  # The cell type shared by the majority of cells
  ind <- which(pr > threshold)
  if (!length(ind)) {return("mixed")}
  rownames(tx)[ind]
})
head(ct)

##      cluster_1    cluster_10    cluster_11    cluster_12    cluster_13
##     "NK cells" "CD8 T-cells"    "NK cells" "CD8 T-cells"   "monocytes"
##     cluster_14
## "B-cells IgM-"

rowData(tse_type)$population_id <- ct[rownames(tse_type)]
rowData(tse_state)$population_id <- ct[rownames(tse_state)]


### Visualization

The cytof_tree tree is considered as a hierarchical structure organizing cell clusters at different granularities. So, an internal node is a cell cluster that incorporates several cell clusters represented by its descendant leaves. Here, we are interested in exploring the expression profile of markers at different resolutions.

**Figure 12.**
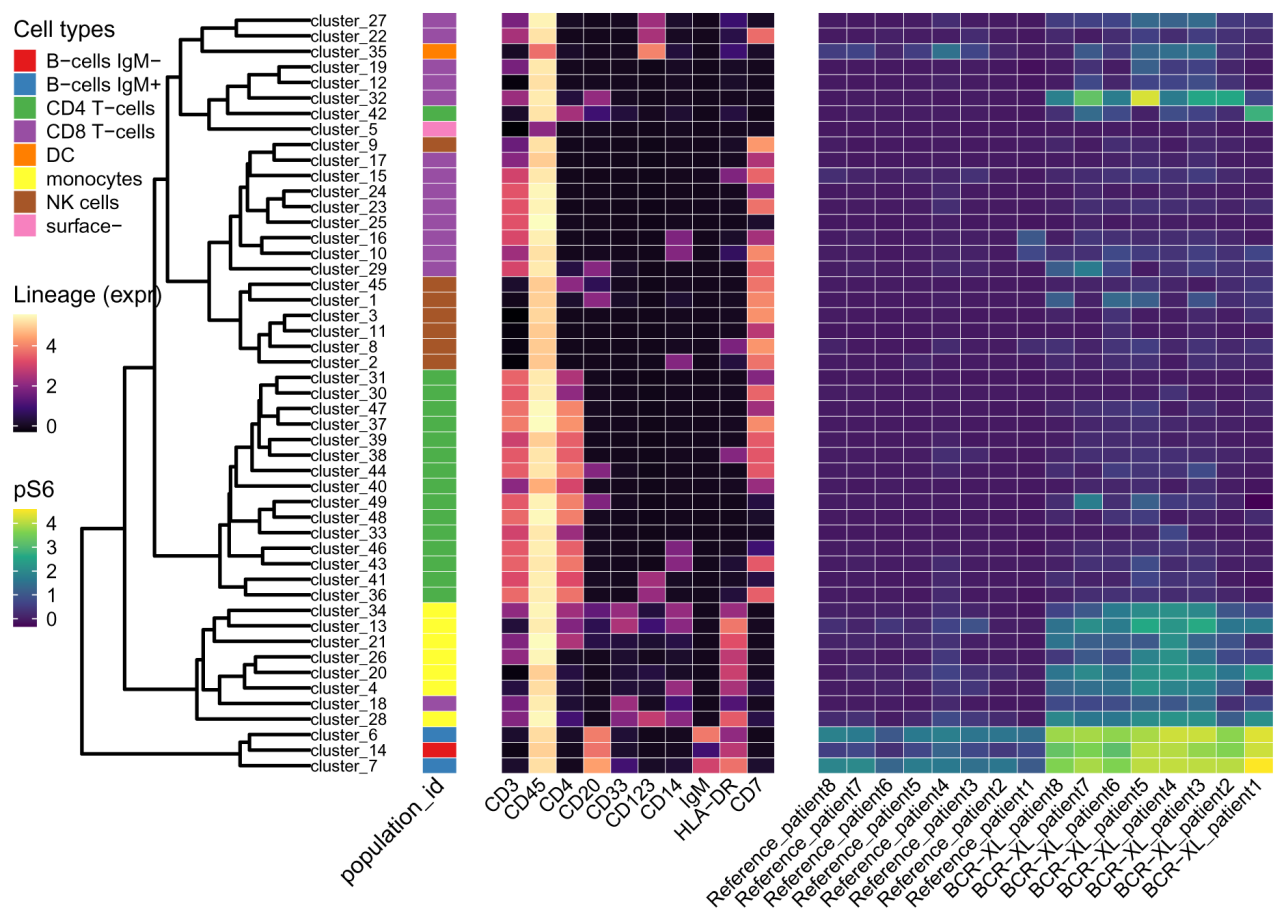
The median expression of markers across 49 cell clusters. Leaves of the tree are labeled with their corresponding cell clusters. Cell types (population_id) of leaves are shown in the first heatmap. Median expressions of ten lineage markers on each leaf are shown in the middle heatmap. Cell clusters in the same branch show similar expression patterns of lineage markers. The right heatmap is about the median expression of a functional marker pS6 in clusters (rows) per sample (column).



We customize a function treeHM (below) to draw a tree along with three heatmaps as Figure 12. The function is created mainly based on ggtree and gheatmap from the R package *ggtree*. The use of different color palettes for heatmaps is enable by scale_fill_* (from *ggplot2*) and new_scale_fill() (from *ggnewscale*).


treeHM <- function(tse_type, tse_state, select = "pS6") {
   # plot the tree
   plot_1 <- ggtree(rowTree(tse_type), ladderize = FALSE) +
    geom_tiplab(size = 1.8, align = TRUE)




# viz cell types of clusters in the 1st heatmap
cluster_type <- rowData(tse_type)[, "population_id", drop = FALSE]
plot_2 <- gheatmap(p = plot_1, data = cluster_type, width = 0.15,
                      offset = 3.5, colnames_angle = 45, hjust = 1,
                      font.size = 2.5) +
  scale_fill_brewer(palette = "Set1", name = "Cell types",
                       guide = guide_legend(order = 1)) +
  new_scale_fill()




# viz expression of lineage markers in the 2nd heatmap
plot_3 <- gheatmap(p = plot_2, data = assays(tse_type)[[1]], width = 1.2,
                      offset = 6.5, colnames_angle = 45, hjust = 1,
                      font.size = 2) +
  scale_fill_viridis_c(option = "A", name = "Lineage (expr)",
                          guide = guide_colourbar(order = 2)) +
  new_scale_fill()




# viz expression of pS6 in the 3rd heatmap
# The expression of pS6 on all (97) nodes of the tree for 16 samples
sse <- tse_state[, colData(tse_state)$marker_name == select]
mat <- assays(sse)[[1]] %>%
  data.frame(check.names = FALSE) %>%
  mutate(sample_id = paste(rowData(sse)$group_id, rowData(sse)$patient_id,
                               sep = "_"),
          cluster_id = rowLinks(sse)$nodeLab) %>%
  arrange(desc(factor(sample_id))) %>%
  pivot_wider(names_from = sample_id, values_from = !!select) %>%
  column_to_rownames(var = "cluster_id")
plot_4 <- gheatmap(p = plot_3, data = mat, offset = 18,
                      width = 2, colnames_angle = 45, hjust = 1,
                      font.size = 2) +
  scale_fill_viridis_c(option = "D", name = select,
                          guide = guide_colourbar(order = 3)) +
  expand_limits(y = -8)
plot_4 +
  theme(legend.key.size= unit(2.5, "mm"),
         legend.spacing.x = unit(1, "mm"),
         legend.spacing.y = unit(1, "mm"),
         legend.text = element_text(size = 6),
         legend.title = element_text(size = 7),
         legend.background = element_rect(fill = NA),
         legend.position=c(0.05, 0.65))
}


treeHM(tse_type, tse_state, "pS6")


In Figure 12, the expression of pS6 appears different mainly in three cell clusters ( cluster_6, cluster_7 and cluster_14) between stimulated and unstimulated samples. These three clusters all belong to B cells. Also, monocytes seem to have slightly higher pS6 in stimulated samples than in unstimulated samples. cluster_18 is labeled as CD8 T-cells, but it is more similar to monocytes in the expression pattern of lineage markers.

**Figure 13.**
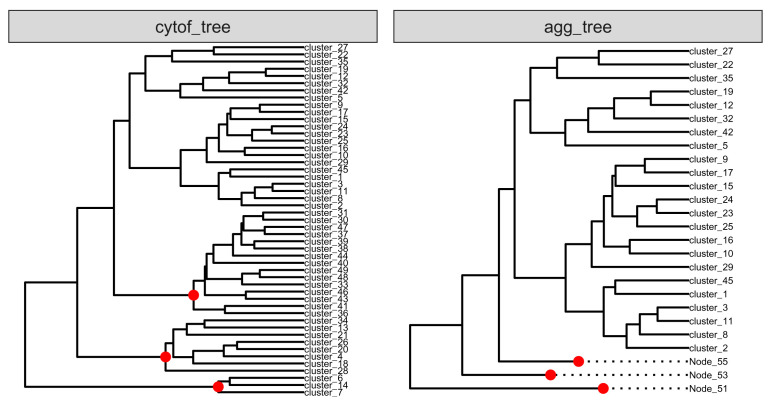
Comparison of two trees: cytof_tree and agg_tree. Three branches that are connected to the three red nodes in cytof_tree are merged, and are presented as dashed lines in agg_tree.

We manipulate cytof_tree by merging its three branches as three internal nodes to creates a new tree agg_tree (see Figure 13). shareNode is used to find the first shared node on paths from specific nodes to the root.


# find branch nodes of the three branches
B_node <- shareNode(tree = cytof_tree, node = c("cluster_6", "cluster_7"))
CD4_node <- shareNode(tree = cytof_tree, node = c("cluster_36", "cluster_31"))
mct_node <- shareNode(tree = cytof_tree, node = c("cluster_34", "cluster_28"))

# Nodes are labeled in red (see Figure)
agg_node <- c(B_node, CD4_node, mct_node)
agg_label <- names(agg_node)

# Set the specific nodes as leaves
agg_tree <- asLeaf(tree = cytof_tree, node = agg_label)

# Generate a figure to compare both trees
both_tree <- list("cytof_tree" = cytof_tree, "agg_tree" = agg_tree)
class(both_tree) <- "multiPhylo"
ggtree(both_tree, ladderize = FALSE) +
  facet_wrap(~.id, scales = "free") +
    geom_tiplab(size = 1.8, align = TRUE) +
geom_point2(aes(subset = label %in% agg_label),
              color = "red", size = 2) +
xlim(c(0, 10))


We replace the rowTree() with the new tree agg_tree, and update Figure 12 to get 14. Also, other functional markers, e.g., pNFkB, can be visualized instead of pS6, which we do not show here.


rowTree(tse_type) <- rowTree(tse_state) <- agg_tree
treeHM(tse_type, tse_state, "pS6")


**Figure 14.**
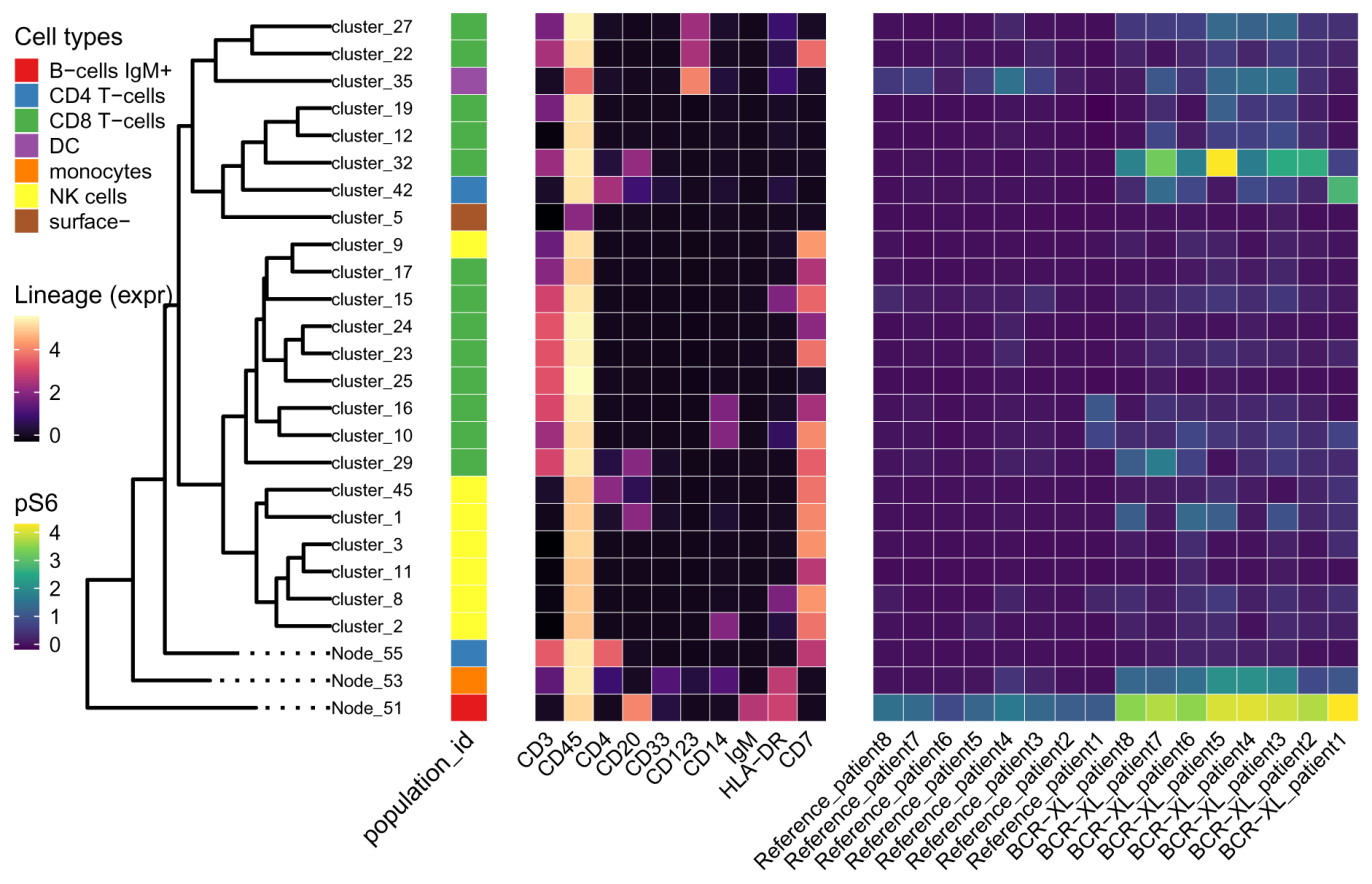
The median expression of markers across 26 cell clusters. This figure is similar to Figure 12 except that B cells (Node_51), monocytes (Node_53) and CD4 T-cells (Node_55) are now visualized at a broader resolution.

Overall, with ***TreeSummarizedExperiment***, single-cell users can over-cluster cells into many cell subpopulations, customize visualization functions to explore data at the high resolution, and finally merge clusters with similar profiles to a suitable resolution to perform downstream analysis.

## Summary

*TreeSummarizedExperiment* is an S4 class in the family of ***SummarizedExperiment*** classes, which enables it to work seamlessly with many other packages in Bioconductor. It integrates the ***SummarizedExperiment*** and the phylo class, facilitating data access or manipulation at different resolutions of the hierarchical structure. By providing additional functions for the phylo class, we support users to customize functions for the ***TreeSummarizedExperiment*** class in their workflows.

## Data availability

### Underlying data

Human Microbiome Project data (v35) and mass cytometry (CyTOF) dataset ^19^ were used for the presented use cases. They can be downloaded using the R package HMP16SData ^14^ and HDCytoData ^20^, respectively.

## Software availability

The TreeSummarizedExperiment package is available at:

https://doi.org/doi:10.18129/B9.bioc.TreeSummarizedExperiment

Source code of the development version of the package is available at:

https://github.com/fionarhuang/TreeSummarizedExperiment

Archived source code as at time of publication: http://doi.org/10.5281/zenodo.4046096 ^23^

## Author information

RH developed the software with contributions from FGME. All authors participated in the conceptualization of the software. RH, CS and MDR drafted the manuscript with review and editing from KCRA, SCH, GY and FGME. All authors read and approved the final manuscript.
