# Supplementary material for: TreeSummarizedExperiment: a S4 class for data with hierarchical structure
Source: F1000Res. 2021 Mar 2;9:1246. Originally published 2020 Oct 15. [Version 2] doi: 10.12688/f1000research.26669.2 (PMC7684683; doi:10.12688/f1000research.26669.2)
Supplement: Peer review history

# Peer review: Reviewer response for version 2

**DOI:** 10.5256/f1000research.54474.r80502

**Published:** 2021-03-19

**Authors:** Shila Ghazanfar

**Affiliations:** 1 Cancer Research UK Cambridge Institute, Li Ka Shing Centre, University of Cambridge, Cambridge, UK

The authors have done an excellent job in addressing each of my questions and implementing feature suggestions where appropriate.

Are the conclusions about the tool and its performance adequately supported by the findings presented in the article?

Yes

Is the rationale for developing the new software tool clearly explained?

Yes

Is the description of the software tool technically sound?

Yes

Are sufficient details of the code, methods and analysis (if applicable) provided to allow replication of the software development and its use by others?

Yes

Is sufficient information provided to allow interpretation of the expected output datasets and any results generated using the tool?

Yes

Reviewer Expertise:

statistics, high throughput genomics data analysis, single cell genomics analysis, spatial gene expression analysis

I confirm that I have read this submission and believe that I have an appropriate level of expertise to confirm that it is of an acceptable scientific standard.

# Peer review: Reviewer response for version 2

**DOI:** 10.5256/f1000research.54474.r80503

**Published:** 2021-03-02

**Authors:** Leo Lahti

**Affiliations:** 1 Department of Computing, Faculty of Technology, University of Turku, Turku, Finland

Approved.

Are the conclusions about the tool and its performance adequately supported by the findings presented in the article?

Yes

Is the rationale for developing the new software tool clearly explained?

Yes

Is the description of the software tool technically sound?

Yes

Are sufficient details of the code, methods and analysis (if applicable) provided to allow replication of the software development and its use by others?

Yes

Is sufficient information provided to allow interpretation of the expected output datasets and any results generated using the tool?

Yes

Reviewer Expertise:

Bioinformatics, open research software, R/Bioconductor, microbiome research, statistical machine learning

I confirm that I have read this submission and believe that I have an appropriate level of expertise to confirm that it is of an acceptable scientific standard.

# Peer review: Reviewer response for version 1

**DOI:** 10.5256/f1000research.29440.r73185

**Published:** 2020-11-23

**Authors:** Matthew Ritchie

**Affiliations:** 1 The Walter and Eliza Hall Institute of Medical Research, Parkville, Vic, Australia

Huang *et al*. describe the TreeSummarizedExperiment package, which provides well-designed S4 infrastructure that couples the phylo and SingleCellExperiment classes to create a container for high-throughput data that can be organised in a tree-like structure. 

 The article is structured like a vignette, providing an overview of the class (Figure 1) and stepping the reader through the process of setting up a TreeSummarizedExperiment object and accessing and assigning data to its various slots, firstly for a toy data set and then for data from the Human Microbiome Project.

 The article is very clearly written, and the authors demonstrate the ability to use TreeSummarizedExperiment objects in conjunction with other established software for dealing with trees (e.g. ggtree and tidyTree) or dimensionality reduction of high-throughout data (e.g. scater). One topic that I was interested to read more about was its potential use in a single cell RNA-seq analysis. Perhaps use cases for such applications can be added as future work. The TreeSummarizedExperiment package has been available from Bioconductor since May 2019 and it has been downloaded > 2.4K times, which indicates it is being taken up by the community.  

 Minor issues:

- Affiliation 5: missing 'O' in 'Oxford'.
- 'Functions operating on the phylo object.' section, sentence 2, missing word: 'such as ape [and] tidytree.'

Are the conclusions about the tool and its performance adequately supported by the findings presented in the article?

Yes

Is the rationale for developing the new software tool clearly explained?

Yes

Is the description of the software tool technically sound?

Yes

Are sufficient details of the code, methods and analysis (if applicable) provided to allow replication of the software development and its use by others?

Yes

Is sufficient information provided to allow interpretation of the expected output datasets and any results generated using the tool?

Yes

Reviewer Expertise:

Transcriptomics (bulk and single cell)

I confirm that I have read this submission and believe that I have an appropriate level of expertise to confirm that it is of an acceptable scientific standard.

# Response

**Published:** 2021-03-02

**Authors:** Ruizhu Huang

**Affiliations:** University of Zürich, Switzerland

1. **Affiliation 5: missing 'O' in 'Oxford'.**
  Thank you.  The typo is fixed.
2. **'Functions operating on the phylo object.' section, sentence 2, missing word: 'such as ape [and] tidytree.'**

**          **The missing word is added now.

# Peer review: Reviewer response for version 1

**DOI:** 10.5256/f1000research.29440.r73184

**Published:** 2020-11-12

**Authors:** Shila Ghazanfar

**Affiliations:** 1 Cancer Research UK Cambridge Institute, Li Ka Shing Centre, University of Cambridge, Cambridge, UK

Huang and colleagues have written a software article presenting TreeSummarizedExperiment [currently version 1.6.0], a Bioconductor package aimed at providing an S4 class for omics data with hierarchical tree structure. The TreeSummarizedExperiment class builds on the popular SingleCellExperiment object class, with additional slots in which hierarchical structure, in the form of phylo objects, can be added for features (rows) and observations (columns). In addition to the object class, the package contains several functions for manipulating these objects, ranging from getting/setting/resetting the tree slots, aggregating across rows and/or columns, and various analytical tasks operating on the phylo objects.

 The article is well written with clear motivation and description of the package, and addresses an important problem of performing analysis of high dimensional hierarchically structured data using object-oriented programming. I have a few further comments and questions that may improve the breadth of use of TreeSummarizedExperiment by the research community. 

- Is there a way to simply include an argument for aggValue() that would swap the order to columns first and rows second, rather than requiring the user to perform two distinct operations?
- The new slots, rowTree, colTree, rowLinks and colLinks are 'getter' accessors but not currently 'setter' functions. I can imagine a popular use-case among users with an already constructed object of class SummarizedExperiment or SingleCellExperiment would be to simply use as(, "TreeSummarizedExperiment") and then attempt to add the additional slots, for example as the output of hclust(). I would suggest prioritising converting these functions to both 'getter' and 'setter', or perhaps adding a constructor usage for TreeSummarizedExperiment for objects that are already SummarizedExperiment or SingleCellExperiment, if possible.
- I'm interested in how TreeSummarizedExperiment would work in the case where the hierarchical structure is not a typical single tree, but comprising of multiple distinct tree structures. An example of such is single cell (or single clone) lineage data where there exists a tree structure within each experimental condition, but not between cells from different conditions. Would the colTree slot correspond to a list of trees in this case?
- How would one go about combining different TreeSummarizedExperiment objects? Do the typical cbind() and rbind() operations have meaning here? In which cases are they not to be used?
- I would be interested in getting to a clustered heatmap as an example of visualisation for the TreeSummarizedExperiment, either implemented using ggplot2/ggtree, or other packages like ComplexHeatmap?
- How would the tree structure information storage scale in terms of the number of rows and columns, or in the hierarchical structures?

Minor/cosmetic

- typo in affiliation 5.
- legend cut off in Figures 6, 7, and 8.

Are the conclusions about the tool and its performance adequately supported by the findings presented in the article?

Yes

Is the rationale for developing the new software tool clearly explained?

Yes

Is the description of the software tool technically sound?

Yes

Are sufficient details of the code, methods and analysis (if applicable) provided to allow replication of the software development and its use by others?

Yes

Is sufficient information provided to allow interpretation of the expected output datasets and any results generated using the tool?

Yes

Reviewer Expertise:

statistics, high throughput genomics data analysis, single cell genomics analysis, spatial gene expression analysis

I confirm that I have read this submission and believe that I have an appropriate level of expertise to confirm that it is of an acceptable scientific standard, however I have significant reservations, as outlined above.

# Response

**Published:** 2021-03-02

**Authors:** Ruizhu Huang

**Affiliations:** University of Zürich, Switzerland

Thank you for your comments!

1. **swap the order to columns first and rows second, rather than requiring the user to perform two distinct operations?**
  aggValue() is now deprecated and replaced by a new function, aggTSE(), that allows users to swap the order of aggregation and define different functions for the row and the column dimension.
2. **The new slots, rowTree, colTree, rowLinks and colLinks are 'getter' accessors but not currently 'setter' functions. I can imagine a popular use-case among users with an already constructed object of class SummarizedExperiment or SingleCellExperiment would be to simply use as(, "TreeSummarizedExperiment") and then attempt to add the additional slots, for example as the output of hclust(). I would suggest prioritising converting these functions to both 'getter' and 'setter', or perhaps adding a constructor usage for TreeSummarizedExperiment for objects that are already SummarizedExperiment or SingleCellExperiment, if possible.**
  rowTree and colTree are now both setters and getters. When the row/column tree is replaced, the rowLinks/colLinks is updated automatically. To avoid breaking links between assays and trees, we don't recommend users to modify the rowLinks/colLinks data. Therefore, rowLinks/colLinks are still kept as getters.
3. **I'm interested in how TreeSummarizedExperiment would work in the case where the hierarchical structure is not a typical single tree, but comprising of multiple distinct tree structures. An example of such is single cell (or single clone) lineage data where there exists a tree structure within each experimental condition, but not between cells from different conditions. Would the colTree slot correspond to a list of trees in this case?**
  Yes, it's possible to have a list of trees in the rowTree/colTree. In the rowLinks/colLinks, we have added a new column (whichTree) to give information about which row/column tree a row/column is mapped to. We have also added a new vignette describing how to combine multiple TSEs. ( https://www.bioconductor.org/packages/devel/bioc/vignettes/TreeSummarizedExperiment/inst/doc/The_combination_of_multiple_TSEs.html)
4. **How would one go about combining different TreeSummarizedExperiment objects? Do the typical cbind() and rbind() operations have meaning here? In which cases are they not to be used?**
  rbind() and cbind() are now implemented for TreeSummarizedExperiment objects. To rbind() multiple TSEs successfully, it's required that the TSEs agree in the column dimension to have the same colTree() and colLinks(). Similarly, cbind() would require TSEs to have the same rowTree() and rowLinks(). More detailed information is available in the new vignette about combining multiple TSEs. ( https://www.bioconductor.org/packages/devel/bioc/vignettes/TreeSummarizedExperiment/inst/doc/The_combination_of_multiple_TSEs.html)
5. **I would be interested in getting to a clustered heatmap as an example of visualisation for the TreeSummarizedExperiment, either implemented using ggplot2/ggtree, or other packages like ComplexHeatmap?**
  We have added a new use case of TSE on CyTOF data, and customized a visualization function based on *ggtree, ggplot2* and *ggnewscale* to plot a clustered heatmap.
6. **How would the tree structure information storage scale in terms of the number of rows and columns, or in the hierarchical structures.**
  We store the tree structure as a phylo object. The size of a phylo object is quite small even for a tree with 10 ^6^ leaves (about 90 Mb). To set up the link between rows/columns to a tree, it takes only a few seconds even for 10 ^6^ rows to a tree with 10 ^6^ leaves.
7. **typo in affiliation 5. legend cut off in Figures 6, 7, and 8.**
  The typo and the legend cut off are fixed.

# Peer review: Reviewer response for version 1

**DOI:** 10.5256/f1000research.29440.r73188

**Published:** 2020-10-19

**Authors:** Leo Lahti

**Affiliations:** 1 Department of Computing, Faculty of Technology, University of Turku, Turku, Finland

This software article introduces TreeSummarizedExperiment, a S4 class for hierarchically structured data in R. This provides a very generic data structure that serves for instance the single cell and microbiome bioinformatics communities, and has already gathered remarkable attention with a growing user base. The package is mature and has been available via Bioconductor for some time already.

 The rationale for developing the new software tool has been clearly explained, and sufficient examples are provided. It extends the popular SingleCellExperiment class structure by bringing in tree info on data rows and cols (based on the phylo class). The new extended class has potentially many new applications, for instance in microbiome research; concrete examples are provided. The new class combines and extends other common class structures, which is very beneficial for the overall compatibility. Many tools for manipulation and use already exist based for instance on related work on the SummarizedExperiment family of classes, phylo tree structure, and the phyloseq class.

 The overall description of the software is technically sound and follows standard conventions in the R/Bioconducor community. Sufficient details have been provided to allow replication of the software development and its use by others; the documentation and examples are sufficient for getting started with and interpreting outputs of the new class for anyone who has the technical skills that are needed to utilize this work.

**Major**

1. Efficiency of the new method could be discussed further; does this  scale up to population level cohorts that have thousands of samples are increasing hierarchical resolutions?
2. How easy it would be to incorporate further supporting information on the rows and columns, for instance on DNA/RNA sequence information?
3. The class is very generic; is the idea that this package can be used as such in (hierarchical) single-cell experiments, microbiome research, and potentially other fields that have little overlap currently? Or is this package meant to be a fundamental structure that can be further extended in more specific application domains? Some more discussion on these aspects could help to contextualize the new class.

**Minor**

1. " ssay_data" -> "assay_data"

Are the conclusions about the tool and its performance adequately supported by the findings presented in the article?

Yes

Is the rationale for developing the new software tool clearly explained?

Yes

Is the description of the software tool technically sound?

Yes

Are sufficient details of the code, methods and analysis (if applicable) provided to allow replication of the software development and its use by others?

Yes

Is sufficient information provided to allow interpretation of the expected output datasets and any results generated using the tool?

Yes

Reviewer Expertise:

Bioinformatics, open research software, R/Bioconductor, microbiome research, statistical machine learning

I confirm that I have read this submission and believe that I have an appropriate level of expertise to confirm that it is of an acceptable scientific standard.

# Response

**Published:** 2021-03-02

**Authors:** Ruizhu Huang

**Affiliations:** University of Zürich, Switzerland

Thank you for reviewing our work.

1. **Efficiency of the new method could be discussed further; does this  scale up to population level cohorts that have thousands of samples are increasing hierarchical resolutions?**
  TreeSummarizedExperiment (TSE) inherits the slots of the SummarizedExperiment (SE) & SingleCellExperiment (SCE) classes, and adds new slots like rowTree, colTree, rowLinks, colLinks, referenceSeq. For operations involving the inherited slots, TSE works similarly as SE and SCE. For the new slots, the data manipulation depends on the functions that users have applied on the tree object (of class phylo). These functions might be from TSE or outside TSE. For functions from TSE, either those working on the phylo tree (e.g., findDescendant, convertNode, matTree, addLabel) or those working on TSE (e.g., rowTree, colTree, rowLinks, colLinks, changeTree), takes only seconds even for a tree with up to 100,000 nodes. 
2. **How easy it would be to incorporate further supporting information on the rows and columns, for instance on DNA/RNA sequence information?**
  TSE now has a slot referenceSeq to store the sequence information of features ( rows).
3. **The class is very generic; is the idea that this package can be used as such in (hierarchical) single-cell experiments, microbiome research, and potentially other fields that have little overlap currently? Or is this package meant to be a fundamental structure that can be further extended in more specific application domains? Some more discussion on these aspects could help to contextualize the new class.**
  Currently, there is not much overlap in the community across fields, e.g, single-cell experiments, microbiome research. But, we do see that they share similarities in data structures, and can potentially share synergies in data visualization or analysis. We provide TSE as a standalone R package like SummarizedExperiment and SingleCellExperiment, and propose it as a convenient starting point to create R packages for downstream analysis or visualization of data with tree structures. We are open to update our work or receive pull requests if new features (or slots) required in a specific field are feasible to be integrated to TreeSummarizedExperiment.   For example, a new optional slot referenceSeq(), which was requested mainly for microbiome data to store RNA/DNA sequencing information, has been developed by Félix G.M. Ernst,  and the PR has been accepted in TreeSummarizedExperiment. 
4. **" ssay_data" -> "assay_data"**
  The typo is fixed.
